# Oral Chinese Herbal Medicine Combined with Pharmacotherapy for Stable COPD: A Systematic Review of Effect on BODE Index and Six Minute Walk Test

**DOI:** 10.1371/journal.pone.0091830

**Published:** 2014-03-12

**Authors:** Xiankun Chen, Brian May, Yuan Ming Di, Anthony Lin Zhang, Chuanjian Lu, Charlie Changli Xue, Lin Lin

**Affiliations:** 1 Evidence-Based Medicine & Clinical Research Service Group, Guangdong Provincial Academy of Chinese Medical Sciences, Guangzhou, China; 2 Guangdong Provincial Hospital of Chinese Medicine, Guangzhou, China; 3 Traditional & Complementary Medicine Research Program, Health Innovations Research Institute, School of Health Sciences, RMIT University, Melbourne, Australia; 4 Guangdong Provincial Academy of Chinese Medical Sciences, Guangzhou, China; 5 Department of Respiratory Medicine, Guangdong Provincial Hospital of Chinese Medicine, Guangzhou, Guangdong Province, China; University of Leicester, United Kingdom

## Abstract

This systematic review evaluated the effects of Chinese herbal medicine (CHM) plus routine pharmacotherapy (RP) on the objective outcome measures BODE index, 6-minute walk test (6MWT), and 6-minute walk distance (6MWD) in individuals with stable chronic obstructive pulmonary disease (COPD). Searches were conducted of six English and Chinese databases (PubMed, EMBASE, CENTRAL, CINAHL, CNKI and CQVIP) from their inceptions until 18th November 2013 for randomized controlled trials involving oral administration of CHM plus RP compared to the same RP, with BODE Index and/or 6MWT/D as outcomes. Twenty-five studies were identified. BODE Index was used in nine studies and 6MWT/D was used in 22 studies. Methodological quality was assessed using the Cochrane Risk of Bias tool. Weaknesses were identified in most studies. Six studies were judged as ‘low’ risk of bias for randomisation sequence generation. Twenty-two studies involving 1,834 participants were included in the meta-analyses. The main meta-analysis results showed relative benefits for BODE Index in nine studies (mean difference [MD] −0.71, 95% confidence interval [CI] −0.94, −0.47) and 6MWT/D in 17 studies (MD 54.61 meters, 95%CI 33.30, 75.92) in favour of the CHM plus RP groups. The principal plants used were *Astragalus membranaceus, Panax ginseng* and *Cordyceps sinensis. A. membranaceus* was used in combination with other herbs in 18 formulae in 16 studies. Detailed sub-group and sensitivity analyses were conducted. Clinically meaningful benefits for BODE Index and 6MWT were found in multiple studies. These therapeutic effects were promising but need to be interpreted with caution due to variations in the CHMs and RPs used and methodological weakness in the studies. These issues should be addressed in future trials.

## Introduction

Chronic obstructive pulmonary disease (COPD) is a disorder characterized by progressive development of airflow limitation and an enhanced chronic inflammatory response in the airways [1]. It is predicted to become the third most frequent cause of death in the world by 2020 [2]. The aim of current routine pharmacotherapy (RP) for stable COPD is to control disease progression by using bronchodilators and anti-inflammatory drugs [1]. However the treatment outcomes remain less than satisfactory and some RPs have been associated with adverse events such as cardiovascular events, oropharyngeal candidiasis and risk of pneumonia [1].

The use of the complementary and alternative medicine (CAM) is relatively common among stable COPD sufferers. One survey in Australia found that 41% of respondents had used some form of CAM to improve their health, counteract side effects of medications and/or compensate for dietary deficiencies [3]. Chinese herbal medicine (CHM) is currently considered a complementary or alternative medicine in most Western countries and has become increasingly accepted worldwide [4]. CHMs are prescribed as single herbs or made into formulae according to the therapeutic actions of each herb and also their effects in combination [5]. Studies have indicated that some plants have anti-inflammatory effects [6]–[9], can improve immune functions [10]–[14] and can assist in expelling phlegm [15], [16]. CHMs can be administered as oral decoctions or prepared into pills, powders or capsules. They are commonly combined with routine pharmacotherapy in an attempt to improve outcomes [17].

A recent review of clinical trials of CHM reported that CHM plus RP had a better effect on quality of life (QoL) for patients with stable COPD compared to RP alone [18]. However, QoL measures largely depend on patients' perception, so the additional benefit of CHM that was reported may have been due to the effect of an additional therapy rather than to the specific effects of the CHMs. Therefore, this review focuses on trials that used more objective outcome measures in order to determine whether adding CHMs to pharmacotherapy produced clinical benefits for patients with stable COPD.

### Six-Minute Walk Test

Objectively measured exercise impairment is reported to be a powerful indicator of health status impairment and a predictor of prognosis [19]. The Six-Minute Walk Test (6MWT) is a well-validated standardised test of exercise capacity that has been used as an outcome measure in clinical trials of COPD, is well tolerated, and reflects everyday life like activities [20].

Change in 6MWT is a predictor of survival in COPD [21] and low values on 6MWT have been shown to be predictive of mortality [22]. It was found that 6MWT was a significant predictor of survival with longer times to death observed in patents with longer walking distances [21]. The minimal clinically important difference for the 6MWT in COPD has been estimated using various methods as 54 meters [23] and more recently as 25 meters [24]. In a 3-year prospective multicentre observational study, the mean six minute walk distance (6MWD) declined by 1.6 meters per year in moderate COPD patients, defined as stage II according to the Global Initiative for Chronic Obstructive Lung Disease (GOLD) classification, 9.8 meters per year in GOLD III, and 8.5 meters per year in GOLD IV [25]. A longer observational study showed that COPD mortality was much higher for patients walking less than 350 meters [26].

### BODE Index

The BODE Index is a multidimensional scoring system that has gained broad acceptance and has been developed as a prognostic marker for COPD patients in an attempt to integrate not only the respiratory but also the systemic expressions of COPD. It is composed of: body mass (B), degree of airflow obstruction (O), level of functional dyspnea (D) and exercise capacity (E) [27]. Body mass is calculated as body mass index (BMI), airflow obstruction is measured as forced expiratory volume in one second (FEV1%) predicted, dyspnoea is measured using the modified Medical Research Council (MMRC) dyspnoea scale, and exercise capacity is measured as 6MWT [27]. Of the BODE Index components, 6MWT was found to have a better predictive ability for mortality than the other three components [28].

In this 10 point scale, higher scores indicate a higher risk of death. For a one-point increase in the BODE Index, the hazard ratio for all-cause mortality was found to be 1.34 [27]. The minimal clinically important difference for the BODE Index has been set at 1 point [29]. Cohort studies have found that the BODE Index had superior predictive power for mortality and hospitalization than the GOLD stage over a median period of 16.2 months [28], and had similar clinical utility to the GOLD stage in predicting the risk for exacerbations during one year [30]. In a long-term study (mean 52 months), BODE Index was found to correlate well with COPD mortality and with scores on the St. George's Respiratory Questionnaire (SGRQ), although BODE Index was the better predictor of mortality [31].

BODE Index has been used in clinical trials as an outcome measure. In a study of the effects of lung volume reduction surgery, post-operative reduction in BODE Index was found to correlate with survival and a decrease to a lower BODE Index quartile was associated with a reduction in mortality (hazard ratio 0.497) [32].

The aim of this review was to evaluate the evidence for efficacy of therapies using CHM plus RP versus the same RP in stable COPD based on RCTs that used the objective outcome measures 6MWT/D and/or BODE Index. In addition, the review aimed to assess the safety of the combined treatments.

## Methods

### Search strategy

The electronic databases PubMed, Cochrane Central Register of Controlled Trials (CENTRAL), EMBASE, CINAHL, China National Knowledge Infrastructure (CNKI) and Chinese Scientific Journals Full text Database (CQVIP) were searched from their inceptions until 18th November, 2013, without language restriction. Search terms were in three groups: condition (COPD, chronic bronchitis and related terms); intervention (CHM, Traditional Medicine and related terms) and study type (Table S1). The three groups of terms were combined and the search results downloaded into Endnote libraries for each data-base. The results for all searches were combined, and duplicates were removed. Relevant review articles were examined to identify any additional studies.

### Inclusion criteria

CHMs were defined as preparations originating from plants or parts of plants (such as seeds, roots, stems, leaves, flowers, fruits, or tubers) that have been used therapeutically in China. Plant-derived purified compounds were not included within this definition of CHM. For example, Tanshinone IIA which is a compound isolated from *Salvia miltiorrhiza* was not considered a CHM [33]. Also, herbs such as Echinacea which is of American origin were considered as beyond the scope of CHM.

Studies included in this review are randomized controlled trials (RCTs) published in English or Chinese that meet all the following criteria: (1) Participants: patients diagnosed with COPD in the stable stage and had no current infection, exacerbation or hospitalization; (2) Types of intervention and control: interventions were orally administered CHM in any form combined with RP (i.e. beta2-agonist, anticholinergic, theophylline, inhaled corticosteroid, mucolytic, or other routine therapy) with the same RP being used in the control arm. Non-pharmacologic respiratory therapy (i.e. smoking cessation, pulmonary rehabilitation, oxygen therapy or pulmonary exercise) was allowed as a co-intervention; (3) Types of outcome measurement: only studies using BODE Index and/or 6MWD/T as outcome measures were considered.

### Exclusion criteria

Clinical trials were excluded if they did not meet the above criteria. In addition, the following types of studies were excluded: (1) Oral CHM used in combination with other non-oral interventions such as acupuncture or injections of a CHM preparation; (2) Studies that included participants with respiratory failure, pulmonary hypertension or *cor pulmonale*; (3) Studies using antibiotics as RP were excluded since antibiotics are recommended for exacerbations and not for stable COPD [34].

### Study selection, data extraction and quality assessment

#### Study selection

One researcher (XC) conducted the searches. The resultant titles and abstracts were screened independently by XC and YD. Irrelevant citations were excluded. The full texts of potentially relevant articles were obtained. Two researchers (XC and YD) independently assessed the eligibility of these articles against the inclusion and exclusion criteria. Issues were resolved by agreement after discussion with a third reviewer (BM).

#### Data extraction and management

Two researchers (XC and YD) extracted data from each study using a standard data extraction form which included details of author, year, country, source of patients, trial design, treatment duration, follow-up period, number of participants, age of patients, severity of COPD, differentiation of syndrome, dropouts, adverse events, and methodological aspects. Attempts were made to contact the original investigators regarding any missing data or to obtain data clarification but no responses were obtained. The extracted data were checked against the full text articles by BM. Any discrepancies were resolved by agreement after rechecking the source papers and further discussion with BM.

#### Assessment of risk of bias in included studies

In accordance with recommendations in the Cochrane Handbook [35], the methodological quality of trials was independently evaluated by two researchers (XC and YD) using the Cochrane risk of bias assessment tool. For each study the following domains were assessed: random sequence generation; allocation concealment; blinding of participants and personnel; blinding of outcome assessors; incomplete outcome data; and selective outcome reporting.

### Data analysis

Data were analysed using Review Manager version 5.2, developed by the Cochrane Collaboration. Mean difference (MD) was used for continuous data and risk ratio (RR) was calculated for dichotomous data, both with 95% confidence intervals (CI). Heterogeneity was tested with I^2^. I^2^ values of 50% or more indicated a substantial level of heterogeneity, so data were pooled using a random effect model (REM). Otherwise, a fixed effect model (FEM) was applied. Sensitivity analysis was used to explore heterogeneity. Possible publication bias was checked using funnel plots when ten or more studies reported the same outcome measure [35]. Egger's test was conducted using STATA 12.0 software (StataCorp LP, USA) to determine whether the funnel plots were symmetrical. Correlation analysis was conducted in IBM SPSS 20.

## Results

### Description of studies

**Figure 1:**
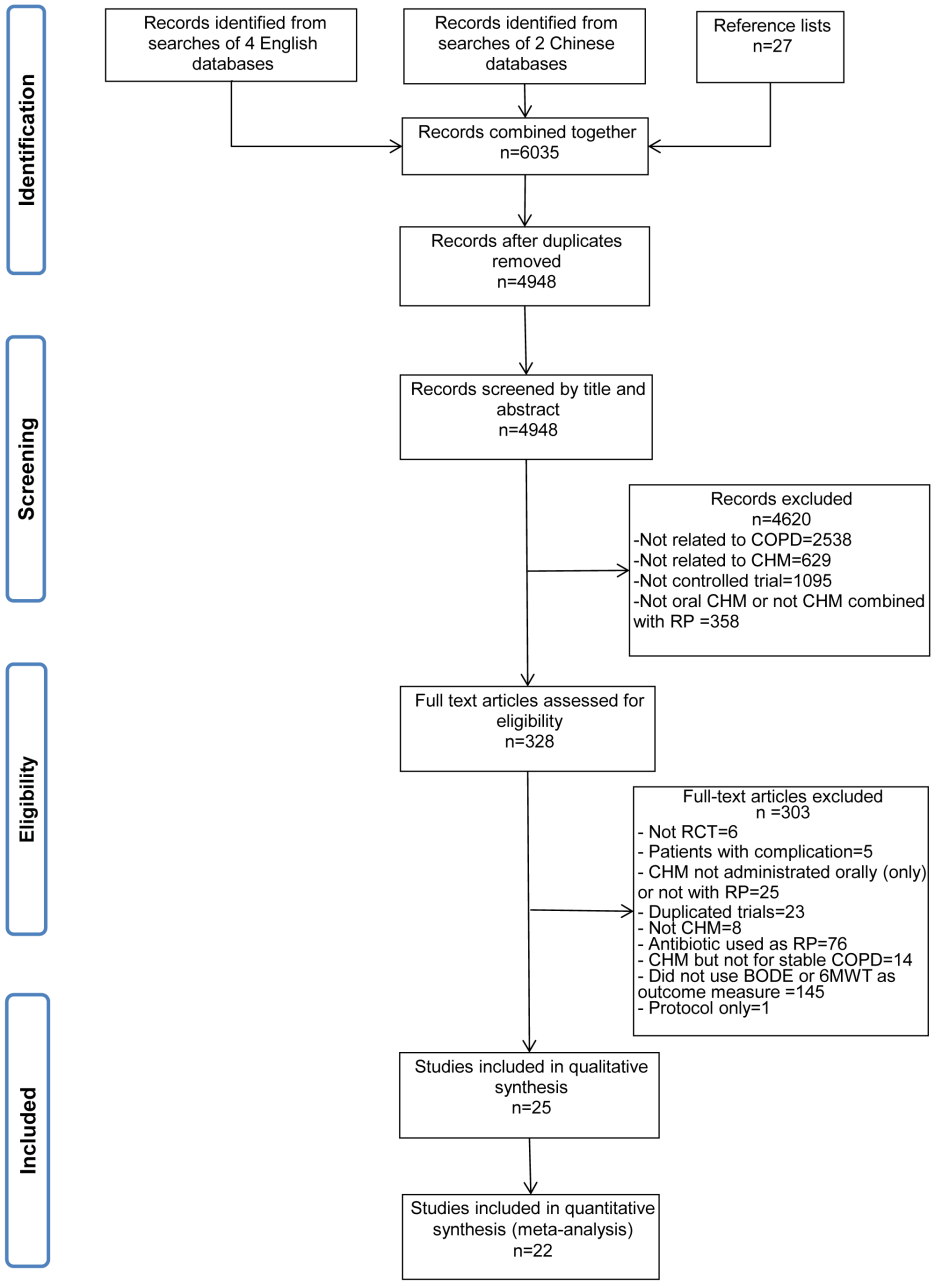
Flowchart of the search and study selection process.

The study selection process is outlined in Figure 1. Of the 4,948 records screened, 328 full text articles were considered and 25 studies met all criteria and were included in this review [36]–[60]. One study was indexed in the English language databases [43], two were indexed in both English and Chinese databases [38], [54] and the other 23 studies were indexed exclusively in Chinese databases [36], [37], [39]–[42], [44]–[53], [55]–[60]. The characteristics of the included studies are shown in Table S2. All the trials were conducted in China from 2004 to 2013. Two trials were reported in English [43], [54]. Treatment duration ranged from 1 month to 12 months (median: 3 months) and 7 out of 25 studies had a treatment duration of six months or more [36], [40], [42], [43], [47], [51], [53]. Five studies mentioned a follow-up period [41], [43], [46], [51], [55]. Two studies had a 6-month follow-up [41], [42] and four had a one-year follow-up [43], [46], [51], [55]. Fifteen studies specified participants' COPD stage [39]–[41], [43], [45], [47], [48], [50], [52], [54]–[58], [60] and 17 studies gave information on the number of years that patients had suffered from COPD [36], [41]–[49], [51]–[53], [55]–[58]. Thirteen studies described the Chinese medicine differentiation of syndrome [38], [40], [43]–[45], [48]–[51], [53], [55], [56], [58]. Nine studies used BODE Index [37], [40], [42], [47], [49], [50], [54], [58], [60] and 22 studies used 6MWT/D [36]–[46], [48], [49], [51]–[59] as outcome measures.

In total, there were 2,385 participants in the 25 studies. Study size ranged from 40 to 352 participants with nine studies having more than 100 participants [39], [40], [43], [46], [51]–[53], [55], [59]. Nine studies reported dropouts during the treatment with or without reasons totalling 54 participants [42], [43], [47], [49], [50], [53], [54], [56], [58]. One study used intention-to-treat analysis (last case carried forward) [43]. Three studies (involving 497 participants) did not provide numerical data suitable for meta-analysis [43], [56], [57], so 22 studies involving 1,834 participants (56% male, age range 36∼83 years) were included in the meta-analyses. Meta-analysis of BODE Index was based on 9 studies [37], [40], [42], [47], [49], [50], [54], [58], [60] and 6MWT/D was based on 17 studies [36]–[39], [41], [44]–[46], [48], [49], [51]–[55], [58], [59], four of which also reported data on BODE Index [37], [49], [54], [58].

### Interventions

The interventions used in the included studies are shown in Table S3.

#### Types of CHM

Six studies used two or three herbal formulae in combination [40], [41], [43], [45], [49], [53], while the others used one formula [36]–[39], [42], [44], [46]–[48], [50]–[52], [54]–[60]. In total, 29 different CHM formulae were used in the form of decoction, capsule or granule. Two studies selected the formulae according to patients' traditional Chinese medicine (TCM) syndrome differentiation [43], [49]. Fourteen studies provided information on the quality control of the herbal ingredients [36], [39]–[46], [51], [54], [55], [58], [59].

The most commonly used formulae were: ‘Bai-Ling Capsule’ aka ‘Jin-Shui-Bao Capsule’ in five studies [40], [41], [45], [46], [51] followed by ‘Yu-Ping-Feng’ decoction or granule in three studies [41], [43], [44]. Both ‘Bai-Ling Capsule’ and ‘Jin-Shui-Bao Capsule’ contained cultured *Cordyceps sinensis* (*Dongchongxiacao*) mycelia [40], [41], [45], [46], [51]. ‘Yu-Ping-Feng’ decoction or granule contained *Astragalus membranaceus (Huangqi)*, *Atractylodes macrocephala (Baizhu)* and *Saposhnikovia divaricata (Fangfeng)*.

Eighty distinct herbs were used in total. The ten most frequently used were: *Astragalus membranaceus* (*Huangqi*) [36]–[38], [40], [41], [43]–[45], [48], [49], [53], [55], [57]–[60], *Panax ginseng* (*Renshen*) [38], [39], [41]–[43], [52], [60], *Atractylodes macrocephala* (*Baizhu*) [36], [37], [41], [43], [44], [47], [48], [55]–[57], [60], *Glycyrrhiza uralensis* (*Gancao*) [36], [38], [47], [48], [50], [53], [55], [57], [60], *Poria cocos* (*Fuling*) [37], [38], [45], [47], [48], [53], *Codonopsis pilosula* (*Dangshen*) [36], [37], [43], [45], [47], [49], [55], [57], [59], *Cordyceps sinensis* (*Dongchongxiacao*) [40], [41], [45], [46], [51], [59], *Citrus tangerina* (*Chenpi*) [2], [26], [31], [55], [57], [60], *Ophiopogon japonicus* (*Maimendong*) [38], [41], [43], [48], [50], [56], and *Epimedium grandiflorum* (*Yinyanghuo*) [43], [48], [54], [55].

*Astragalus membranaceus* (*Huangqi*) was used in combination with other herbs in 18 formulae in 16 studies [36]–[38], [40], [41], [43]–[45], [48], [49], [53], [55], [57]–[60]. Ten of these formulae were decoctions [36]–[38], [45], [48], [53], [55], [57], [58], [60], seven were granules [41], [43], [44], [49], [59], and one was made into capsules [40]. The most frequently used herbal combination was *A. membranaceus* plus *A. macrocephala* which was used in ten formulae [36], [37], [41], [43], [44], [48], [55], [57], [60].

#### Types of routine pharmacotherapy (RP) and other routine therapy

Three studies did not provide detailed information about the routine treatment [44], [54], [59]. Two of these mentioned pulmonary rehabilitation in addition to unspecified routine pharmacotherapy [54], [59]. Comprehensive pulmonary rehabilitation programs generally have four major components: exercise training, education, psychosocial/behavioural intervention, and outcome assessment [61]. In 7 studies [41], [43], [45], [48]–[50], [55], patients were treated based on severity according to the recommendations of the GOLD guideline or the Guideline of the Chinese Society of Respiratory Disease (CSRD) [62]. Fifteen studies specified the medications used [36]–[40], [46], [47], [51]–[53], [56]–[60]. Inhalation of ipratropium bromide was used in three studies [38], [42], [51] one of which also used oxygen therapy [38]. Salbutamol was used alone in one study [40], and combined with ipratropium and pulmonary exercise in one study [36]. Four studies used inhalation of salmeterol plus fluticasone [37], [53], [56], [60]. Theophylline was used orally alone in one study [47], in combination with pulmonary rehabilitation in one study [58], with inhaled salmeterol plus fluticasone in one study [57], and with oral Mucosolvan and inhaled long-acting β2 agonists in two studies [39], [52]. One study used a compound oral medication which contained aminophylline, bromhexine and chlorphenamine [46].

### Risk of bias

Risk of bias assessment details are provided in Table 1. All 25 studies were described by the original authors as ‘randomised’. Three of these studies reported that the random sequence was generated by a random number table [36], [42], [44], one used a ‘stratified and block randomization design’ with detailed information on the sequence generation [43], one used ‘drawing of lots’ [46] and one used a ‘computer random number generator’ [58]. For allocation concealment, one study used ‘sealed envelopes’ [43] which was judged as low risk of bias, while the remainder did not describe this aspect [36]–[42], [44]–[60]. One study specified that the randomisation process was conducted by an independent person [43].

**Table 1:** Assessment of risk of bias for 19 studies based on the Cochrane Handbook.

	Sequence Generation	Allocation Concealment	Blinding of Participants	Blinding of Personnel	Blinding of Outcome Assessment	Incomplete Outcome Data	Selective Outcome Reporting
Chen, 2009 [37]	U	U	U	U	U	U	L
Chen, 2012[36]	L	U	U	U	U	L	L
Cui, 2004 [38]	U	U	U	U	U	L	L
Guo, 2008 [39]	U	U	U	U	U	L	L
Hu, 2012 [40]	U	U	U	U	U	L	L
Huang, 2005 [41]	U	U	U	U	U	L	L
Jian, 2012 [42]	L	U	U	U	U	L	L
Li, 2012 [43]	L	L	H	H	L	L	H
Liao, 2011 [44]	L	U	U	U	U	L	L
Liu, 2009 [45]	U	U	U	U	U	L	L
Mao, 2009 [46]	L	U	U	U	U	U	L
Shan, 2011 [47]	U	U	U	U	U	L	L
Xu(2), 2012 [48]	U	U	U	U	U	L	L
Xu (1), 2012 [49]	U	U	U	U	U	U	L
Yu, 2011 [50]	U	U	U	U	U	U	L
Zhang (2), 2007 [51]	U	U	U	U	U	L	L
Zhang (1), 2007 [52]	U	U	U	U	U	L	L
Zhang, 2011 [53]	U	U	U	U	U	L	L
Zhao, 2012 [54]	U	U	U	U	H	L	L
Fan, 2012 [55]	U	U	U	U	U	L	L
Liang, 2013 [56]	U	U	U	U	U	L	H
Lin, 2013 [57]	U	U	U	U	U	L	H
Peng, 2013 [58]	L	U	U	U	U	L	L
Yang, 2013 [60]	U	U	U	U	U	L	L
Zeng, 2013 [59]	U	U	U	U	U	L	L

Risk of Bias Judgements: L: Low risk, U: Unclear risk, H: High risk.

Four studies mentioned that they were single blind [44], [45], [50], [54], with one study using a CHM placebo [54]. However, blinding of participants and personnel were judged as unclear risk of bias for these four studies as they had no descriptions of the blinding procedures. There was one ‘open-label’ study [43], which was judged as high risk of bias for blinding of participants and personnel. The other twenty studies did not mention any blinding method for participants or personnel [36]–[42], [46]–[49], [51]–[53], [55]–[60]. One study claimed independent assessors [43]. Another study specified that data were collected by an unblinded investigator [54]. The remaining studies did not provide information on the binding of outcome assessors [36]–[42], [44]–[53], [55]–[60].

There was no withdrawal in fourteen studies [36], [38]–[41], [44], [45], [48], [51], [52], [55], [57], [59], [60]. Seven studies reported withdrawals with reasons [42], [43], [47], [53], [54], [56], [58], and one of these applied intention-to-treat analysis [43]. Two studies reported withdrawals without reasons [49], [50] and two did not provide information [37], [46]. Three studies provided data which were not suitable for meta-analysis, so were judged as high risk of bias for selective outcome reporting [43], [56], [57].

### Publication bias

The number of studies reporting BODE Index was less than ten, so a funnel plot was not applicable. For 6MWT/D studies, as seen in Figure S1, the funnel plot is not symmetric and there are outliers on both sides of the distribution. Egger's test was not significant (t = 1.42, 95% CI −2.15 to 10.75, p = 0.176) suggesting that the risk of publication bias is not high.

### Correlation analysis

**Figure 2:**
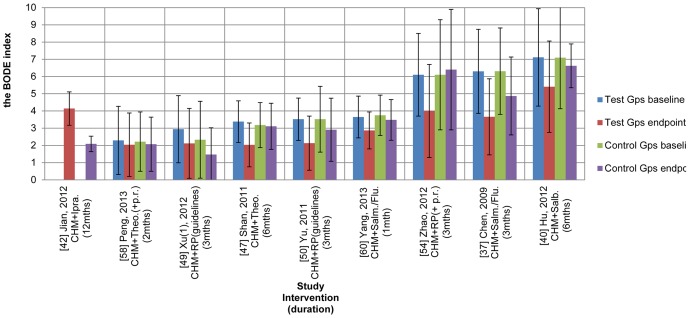
BODE Index mean and SD scores at baseline and end of treatment for 9 studies of CHM plus RP for stable COPD (showing type of intervention). Legend: SD: standard deviation, CHM: Chinese Herbal Medicine, RP: routine pharmacotherapy, mths: months, Gps: groups, Ipra.: ipratropium (inhaled), Theo.(+p.r.): oral theophylline (plus pulmonary rehabilitation), RP (guidelines): routine pharmacotherapy (adjusted for severity according to guidelines), Theo.: theophylline (oral), Salm./Flu.: salmeterol/fluticasone (inhaled), RP(+p.r.): routine pharmacotherapy (pharmacotherapy plus pulmonary rehabilitation), Salb.: salbutamol (inhaled).

**Figure 3:**
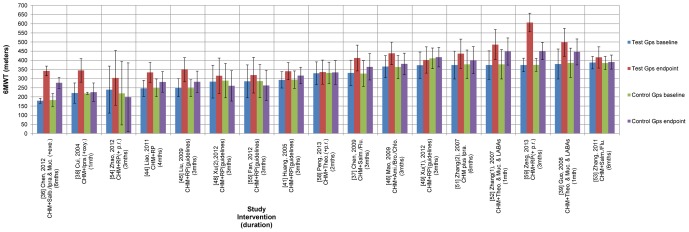
Mean 6MWT/D (meters) with SDs at baseline and end of treatment for 17 studies of CHM plus RP for stable COPD (showing type of intervention). Legend: SD: standard deviation, CHM: Chinese Herbal Medicine, RP: routine pharmacotherapy, mths: months, Gps: groups, Salb./Ipra.: salbutamol/ipratropium (inhaled), exe.: pulmonary exercise, Ipra.: ipratropium (inhaled), oxy.: oxygen therapy, RP(+p.r.): routine pharmacotherapy (pharmacotherapy plus pulmonary rehabilitation), RP (guidelines): routine pharmacotherapy (adjusted for severity according to guidelines), Theo.(+p.r.): oral theophylline (plus pulmonary rehabilitation), Salm./Flu.: salmeterol/fluticasone (inhaled), Ami./Bro./Chlo.: compound oral medication which contained aminophylline, bromhexine and chlorphenamine, Theo.: theophylline (oral), Muc.: Mucosolvan (oral), LABAs: long-acting β2 agonists (inhaled), Salb.: salbutamol (inhaled).

In COPD, there are different levels of severity and patients' conditions tend to deteriorate over time (Figure 2, Figure 3). These factors may affect meta-analyses. To investigate any correlations between severity at baseline and change at the end of treatment (EoT), data were grouped by duration and by treatment or control group and weighted by number of participants in SPSS. In order to focus on the effects of the CHMs and the pharmacotherapies, correlation analysis was conducted after excluding the studies that applied pulmonary rehabilitation as a co-intervention in addition to the RP [54], [58], [59].

For the test groups in the studies that used BODE Index, a negative correlation was found for the three 3-month studies (n = 91) (r = −0.988, p<0.001, two tailed) and for the two 6-month studies (n = 80) (r = −1.000, p<0.001, two tailed). Overall, for the seven studies (n = 225), the higher the BODE Index was at baseline for the CHM plus RP groups, the greater was the benefit gained at the end of treatment (r = −0.707, p<0.001, two tailed). For the control groups, there was a significant negative correlation for the two 6-month studies (n = 78) (r = −1.000, p<0.001, two tailed), for the 3-month studies (n = 88) (r = −0.867, p<0.001, two tailed) and for all seven studies (n = 206) (r = −0.345, p<0.001, two tailed). Therefore there was a greater relative improvement when the patients' COPD was more severe at baseline.

For the three 6-month studies that used 6MWT/D, there was a significant negative correlation for the test groups (n = 143) (r = −0.979, p<0.001, two tailed) and for the control groups (n = 145) (r = −0.985, p<0.001, two tailed). For the 9 studies of 3 to 4 months duration, there was a negative correlation for the test groups (n = 332) (r = −0.31, p = 0.571, two tailed) and for the control groups (n = 324) (r = −0.145, p = 0.09, two tailed). For the 3 studies of one month duration, there was a significant negative correlation for the test groups (n = 130) (r = −0.741, p<0.001, two tailed) but a positive correlation for the control groups (n = 130) (r = 0.963, p<0.001, two tailed). Overall, for the test groups, the longer the distance walked at baseline, the less the benefit conferred by the combined CHM plus RP intervention (n = 605, r = −0.278, p<0.001, two tailed). The same effect was evident for the RP control groups (n = 599, r = −0.115, p = 0.005, two tailed).

### Meta-analyses of outcomes and sensitivity analyses

For the two outcome measures, studies were grouped according to the type of RP used and the duration of the study in order to enable more valid comparisons. Data were pooled when possible. Comparisons between groups at baseline and EoT and within-group differences from baseline to EoT were all investigated.

#### BODE Index

**Figure 4:**
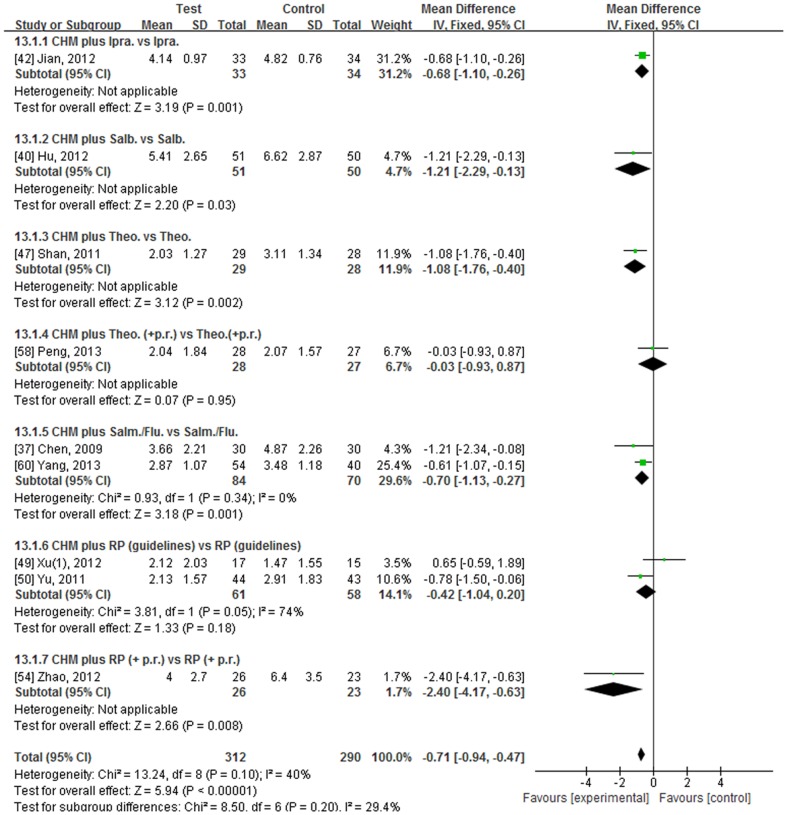
Forest plot comparing CHM plus RP group (Test) to RP group (Control) in terms of BODE Index scores in stable COPD at end of treatment. Legend: CHM: Chinese Herbal Medicine, RP: routine pharmacotherapy, SD: standard deviation, CI: confidence interval, Ipra.: ipratropium (inhaled), Salb.: salbutamol (inhaled), Theo.: theophylline (oral), Theo.(+p.r.): oral theophylline (plus pulmonary rehabilitation), Salm./Flu.: salmeterol/fluticasone (inhaled), RP (guidelines): routine pharmacotherapy (adjusted for severity according to guidelines), RP(+p.r.): routine pharmacotherapy (pharmacotherapy plus pulmonary rehabilitation).

The BODE Index was used in nine studies [37], [40], [42], [47], [49], [50], [54], [58], [60]. Seven studies provided the mean and standard deviation (SD). Two presented data as the number of patients for each BODE Index score [47], [60], so the data were transformed into mean and SD to enable meta-analysis. One study did not provide the baseline data but complete data for the other eight were available [37], [40], [47], [49], [50], [54], [58], [60]. Six studies used a clearly defined RP [37], [40], [42], [47], [58], [60]. The BODE Index scores and SDs are presented in Figure 2 and numerical results for the meta-analyses are presented in Figure 4, Table 2 and Table S4.

**Table 2:** Meta-analysis of BODE Index at end of treatment and sensitivity analysis.

CHM plus RP groups versus the RP groups	BODE score (MD+95%CI) Model
Pooled data for two studies of CHM+Sal./Flu.[37], [60]	−0.70 [−1.13, −0.27], I^2^ = 0% FEM*
	−0.70 [−1.13, −0.27], I^2^ = 0% REM*
Pooled data for four 3-mth studies[37], [49]–[51]	−0.76 [−1.28, −0.24], I^2^ = 66% FEM*
	−0.83 [−1.81, −0.16], I^2^ = 66% REM
Pooled data for two 6-mth studies[40], [47]	−1.12 [−1.69, −0.54], I^2^ = 0%FEM*
	−1.12 [−1.69, −0.54], I^2^ = 0%REM*
Pooled data for 5 studies of AM formulae[37], [40], [49], [58], [60]	−0.54 [−0.89, −0.19], I^2^ = 55.5% FEM*
	−0.51 [−1.07, −0.05], I^2^ = 55.5% REM
Pooled data for all 9 studies[37], [40], [42], [47], [49], [50], [54], [58], [60]	−0.71 [−0.94, −0.47], I^2^ = 40% FEM*
	−0.72 [−1.06, −0.39], I^2^ = 40% REM*
Pooled data for 8 studies[37], [40], [42], [47], [49], [50], [58], [60] after excluding the English-language study[54]	−0.68 [−0.91, −0.44], I^2^ = 27% FEM*
	−0.67 [−0.97, −0.38], I^2^ = 27% REM*
Pooled data for 8 studies exclusively indexed in Chinese databases [37], [40], [42], [47], [49], [50], [58], [60]	−0.68 [−0.91, −0.44], I^2^ = 27% FEM*
	−0.67 [−0.97, −0.38], I^2^ = 27% REM*
Pooled data for 7 studies[37], [40], [42], [47], [49], [50], [60] after excluding studies of pulmonary rehabilitation[54], [58]	−0.72 [−0.97, −0.48], I^2^ = 20% FEM*
	−0.73 [−1.02, −0.44], I^2^ = 20% REM*
Pooled data for 6 studies[37], [40], [42], [47], [58], [60] after excluding studies of undefined RP[49], [50], [54]	−0.72 [−0.97, −0.47], I^2^ = 2% FEM*
	−0.72 [−0.98, −0.46], I^2^ = 2% REM*
Pooled data for 3 studies in which mean BODE was greater than 4 at baseline[37], [40], [54]	−1.40 [−2.12, −0.69], I^2^ = 0% FEM*
	−1.40 [−2.12, −0.69], I^2^ = 0% REM*
Pooled data for 5 studies in which mean BODE was less than 4 at baseline[47], [49], [50], [58], [60]	−0.59 [−0.90, −0.29], I^2^ = 47% FEM*
	−0.53 [−0.98, −0.07], I^2^ = 47% REM*

*Significant difference, CHM: Chinese Herbal Medicine, RP: routine pharmacotherapy, MD: mean difference, CI: confidence interval, mth: month, REM: random effect model, FEM: fixed effect model, AM: Astragalus membranaceus, Salm./Flu.: salmeterol/fluticasone (inhaled).

The longest study employed ipratropium (inhaled) for 12 months but the lack of baseline data meant that it could not be determined whether the BODE Index declined or improved over this period. At EoT there was a significant benefit for the CHM plus ipratropium group of −0.68 points compared to ipratropium alone (Table S4) [42].

Two studies were conducted for 6 months [40], [47]. One study, used salbutamol (inhaled) in patients with a mean BODE Index of 7.1. There was neither improvement nor decline in the salbutamol only group but there was a clinically significant improvement of −1.70 in the group that used *Cordyceps sinensis* mycelia plus salbutamol [40]. The other study used oral theophylline in participants with a much lower mean BODE Index at baseline (T: 3.38, C: 3.18). No significant change was found in the theophylline group at the end of 6 months but a significant benefit was found in the CHM plus theophylline group of −1.35. At EoT there was a clinically significant reduction in BODE Index in favour of the CHM plus theophylline group (MD −1.08) [47] (Figure 4, Table S4).

In the study that used salmeterol/fluticasone (inhaled) for 3 months, the mean BODE Index was 6.31 in the RP group at baseline and there was a significant decrease at EoT (MD −1.44). In the test group, the addition of the CHM produced an improvement of −2.64 over the baseline mean of 6.30 points. At EoT, there was a clinically significant difference between groups of −1.21 in favour of CHM plus salmeterol/fluticasone [37]. Another study also used salmeterol/fluticasone as the RP but the treatment duration was only 1 month. After 1 month there was a non-significant decrease (MD −0.27) in the RP group and a significant decrease in the CHM plus RP group (MD −0.78). At EoT there was a significant difference between groups in favour of the CHM plus RP group (MD −0.61) [60]. There was a significant decrease in the pooled data (MD −0.70) for the two studies in this subgroup [37], [60] (Figure 4, Table 2).

Two studies used RP adjusted according to the guidelines for 3 months [49], [50]. In one study, both groups had a mean BODE Index of 3.52 at baseline and there was a non-significant decline in the RP group at EoT. In the CHM plus RP group, there was a significant decline (MD −1.39), so there was a significant difference between groups at 3 months (MD −0.78) [50]. In the other study, there was a difference in baseline BODE Index mean scores (T: 2.94, C: 2.33) but this was not significant (MD 0.61). After 3 months there were non-significant declines in BODE Index in the RP group and in the CHM plus RP group [49] (Table S4). There was no difference between groups in the pooled data but the heterogeneity was high (Figure 4).

One study used a placebo CHM combined with unspecified RP plus pulmonary rehabilitation (p.r.) in the control group. The baseline mean BODE Index was 6.1 in each group. After three months the RP group remained relatively stable (MD 0.30) but there was a significant improvement in the CHM plus RP group (MD −2.10). At EoT there was a clinically significant benefit in favour of the CHM plus RP group (MD −2.40) [54] (Figure 4, Table S4).

One study used oral theophylline combined with p.r. in patients with a mean BODE Index of 2.2. After two months, there were non-significant decreases in BODE Index in the control group (MD −0.15) and in the test group (MD −0.25). There was no difference between groups at EoT [58] (Figure 4, Table S4).

Based on study duration, the pooled data for the four studies that used CHM plus RP for 3 months showed a decrease in BODE Index from baseline of −1.59 (95%CI −2.06, −1.13, I^2^ = 43%, FEM) [37], [49]–[51]. For the two studies that lasted 6 months, the change was −1.44 (95%CI −1.99, −0.90, I^2^ = 0%, FEM) [40], [47]. The between-groups results were −0.83 for 3 months and −1.12 points for 6 months in favour of the CHM plus RP groups, both of which were significant (Table 2).

For all nine studies, the use of CHM plus RP was associated with a significant decrease in the BODE Index compared with RP alone (MD −0.71, 95%CI −0.94, −0.47, I^2^ = 40%, FEM) [37], [40], [42], [47], [49], [50], [54], [58], [60] (Figure 4). Sensitivity analysis using REM yielded nearly identical results (Table 2). After excluding the single study that was indexed in PubMed a significant decline (MD −0.68) remained, with lower heterogeneity (I^2^ = 27%) [54]. The two studies [54], [58] that used p.r. in addition to RP were excluded from the meta-analysis, since p.r. has been reported to independently improve BODE Index in a controlled study [63] so it may have been a major contributor to the pooled result. The resultant MD was −0.72 (95%CI −0.97, −0.48) and I^2^ was reduced to 20% (FEM). Analysis of data only from the six studies with clearly defined RP also found a similar result (MD −0.86) with low heterogeneity (Table 2) [37], [40], [42], [47], [58], [60]. For the three studies of patients whose mean BODE Index was greater than 4 at baseline, there was a clinically significant benefit for CHM plus RP of −1.40 points (95%CI −2.12, −0.69, I^2^ = 0%, FEM) [37], [40], [54]. For the five studies of less severe patients, whose mean scores were less than 4 at baseline, there was less benefit (MD −0.59) (Table 2) [47], [49], [50], [58], [60].

Regarding the CHMs used in the test groups, five contained *A. membranaceus* (AM) [37], [40], [49], [58], [60]. Compared to baseline, the BODE Index reduced by 1.18 points (95%CI −1.91, −0.44, I^2^ = 68%, REM) in the AM plus RP groups and −0.43 points (95%CI −0.80, −0.05, I^2^ = 0%, REM) in the RP groups. At EoT there was a relative benefit of −0.54 points (95%CI −0.89, 0.19, I^2^ = 48% REM) in favour of the AM plus RP groups (Table 2).

#### 6MWT/D

**Figure 5:**
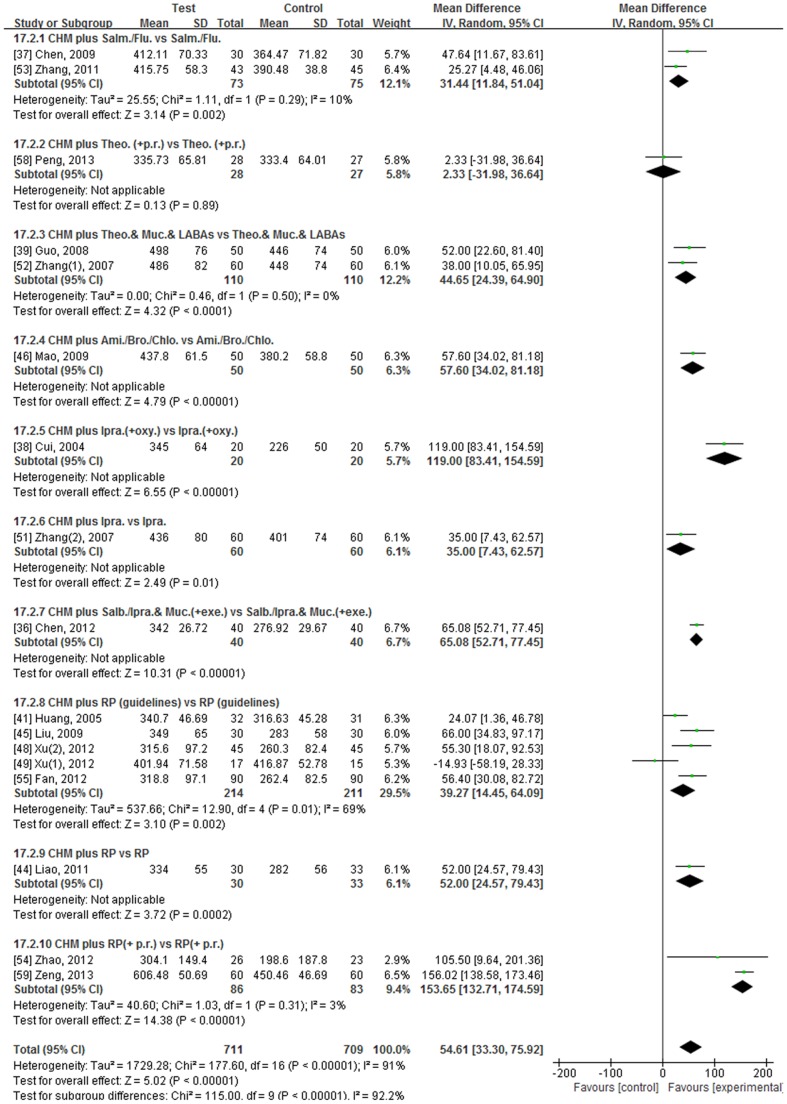
Forest plot comparing CHM plus RP group (Test) to RP group (Control) in terms of 6MWT/D (meters) in stable COPD at end of treatment. Legend: CHM: Chinese Herbal Medicine, RP: routine pharmacotherapy, SD: standard deviation, CI: confidence interval, Salm./Flu.: salmeterol/fluticasone (inhaled), Theo.(+p.r.): oral theophylline (plus pulmonary rehabilitation), Muc.: Mucosolvan (oral), LABAs: long-acting β2 agonists (inhaled), Ami./Bro./Chlo.: compound oral medication which contained aminophylline, bromhexine and chlorphenamine, Ipra.: ipratropium (inhaled), oxy.: oxygen therapy, Salb./Ipra.: salbutamol/ipratropium (inhaled), exe.: pulmonary exercise, RP (guidelines): routine pharmacotherapy (adjusted for severity according to guidelines), RP(+p.r.): routine pharmacotherapy (pharmacotherapy plus pulmonary rehabilitation).

Twenty-two studies used the 6MWT/D [36]–[46], [48], [49], [51]–[59] but the data in five studies were inadequate for meta-analysis [40], [42], [43], [56], [57]. The mean 6MWD and SDs are presented in Figure 3 and numerical results for the meta-analyses are in Figure 5, Table 3 and Table S5.

**Table 3:** Meta-analysis of 6MWT/D (meters) at end of treatment and sensitivity analysis.

CHM plus RP groups versus the RP groups	6MWT meters (MD+95%CI) Model
Pooled data for three 1-mth studies[38], [39], [52]	62.84 [45.23, 80.44], I^2^ = 85% FEM*
	68.49 [22.91, 114.07], I^2^ = 85% REM*
Pooled data for ten 3/4-mth studies[37], [41], [44]–[46], [48], [49], [54], [55], [59]	74.20 [65.42, 82.98], I^2^ = 93% FEM*
	59.66 [24.63, 94.69],I^2^ = 93% REM*
Pooled data for three 6-mth studies[36], [51], [53]	52.12 [42.20, 62.04], I^2^ = 83% FEM*
	43.10 [15.16, 71.04],I^2^ = 83% REM*
Pooled data for 2 studies of CHM+Sal./Flu.[37], [53]	30.87 [12.87, 48.87], I^2^ = 10% FEM*
	31.44 [11.84, 51.04], I^2^ = 10% REM*
Pooled data for 2 studies of CHM+Theo.& Muc.& LABA[39], [52]	44.65 [24.39, 64.90], I^2^ = 0% FEM*
	44.65 [24.39, 64.90], I^2^ = 0% REM*
Pooled data for 5 studies of CHM+RP (guidelines)[41], [45], [48], [49], [55]	40.22 [26.93, 53.50], I^2^ = 69% FEM*
	39.27 [14.45, 64.09], I^2^ = 69% REM*
Pooled data for 2 studies of CHM+RP (+p.r.)[54], [59]	154.40 [137.25, 171.56], I^2^ = 3% FEM*
	153.65 [132.71, 174.59], I^2^ = 3% REM*
Pooled data for 12 studies of AM formulae[36]–[38], [41], [44], [45], [48], [49], [53], [55], [58], [59]	66.21 [59.40, 73.03], I^2^ = 93% FEM*
	55.39 [27.29, 83.50], I^2^ = 93% REM*
Pooled data for 2 studies of Ginseng formulae[39], [52]	44.65 [24.39, 64.90], I^2^ = 0% FEM*
	44.65 [24.39, 64.90], I^2^ = 0% REM*
Pooled data for 5 studies of CS formulae[41], [45], [46], [51], [59]	82.96 [72.63, 93.29],I^2^ = 96% FEM*
	68.11 [12.49, 123.72],I^2^ = 96% REM*
Pooled data for 7 studies in which 6MWD was less than 300 meters at baseline [36], [38], [41], [44], [45], [48], [55]	37.69 [27.25, 48.14], I^2^ = 46% FEM*
	37.05 [22.46, 51.64], I^2^ = 46% REM*
Pooled data for 7 studies in which 6MWD was more than 300 meters at baseline [37], [39], [46], [49], [51]–[53]	59.79 [51.27, 68.30], I^2^ = 72% FEM*
	60.73 [42.62, 78.84], I^2^ = 72% REM*
Pooled data for All 17 studies[36]–[39], [41], [44]–[46], [48], [49], [51]–[55], [58], [59]	62.36 [56.30, 68.42], I^2^ = 91% FEM*
	54.61 [33.30, 75.92], I^2^ = 91% REM*
Pooled data for 16 studies written in Chinese [36]–[39], [41], [44]–[46], [48], [49], [51]–[53], [55], [58], [59]	62.19 [56.11, 68.26], I^2^ = 92% FEM*
	53.10 [31.42, 74.79], I^2^ = 92% REM*
Pooled data for 15 studies exclusively indexed in Chinese databases[36], [37], [39], [41], [44]–[46], [48], [49], [51]–[53], [55], [58], [59]	60.48 [54.32, 66.65], I^2^ = 92% FEM*
	48.99 [26.85, 71.12], I^2^ = 92% REM*
Pooled data for 14 studies[36]–[39], [41], [44]–[46], [48], [49], [51]–[53], [55] after excluding studies of pulmonary rehabilitation[54], [58], [59]	50.96 [44.36, 57.56], I^2^ = 70% FEM*
	48.86 [36.07, 61.64], I^2^ = 70% REM*
Pooled data for 12 studies[37], [39], [41], [44]–[46], [48], [49], [51]–[53], [55] after excluding studies of oxygen [38]& exercise [36] & pulmonary rehabilitation [54], [58], [59]	41.62 [33.63, 49.62], I^2^ = 41% FEM*
	42.05 [31.46, 52.63], I^2^ = 41% REM*
Pooled data for 6 studies[37], [39], [46], [51]–[53] after excluding studies of oxygen [38]& exercise [36] & pulmonary rehabilitation [54], [58], [59] & undefined RP [41], [44], [45], [48], [49], [55]	40.95 [30.19, 51.71], I^2^ = 0% FEM*
	40.95 [30.19, 51.71], I^2^ = 0% REM*

*Significant difference, CHM: Chinese Herbal Medicine, RP: routine pharmacotherapy, MD: mean difference, CI: confidence interval, mth: month, REM: random effect model, FEM: fixed effect model, Salm./Flu.: salmeterol/fluticasone (inhaled), Theo.: theophylline (oral), Muc.: Mucosolvan (oral), LABAs: long-acting β2 agonists (inhaled), RP(guidelines): routine pharmacotherapy (adjusted for severity according to guidelines), RP(+p.r.): routine pharmacotherapy (pharmacotherapy plus pulmonary rehabilitation), AM: *Astragalus membranaceus*, CS: *Cordyceps sinensis*.

When studies were pooled according to duration, for the 3 studies of one month the relative benefit for the addition of the CHM was 68.49 meters (95%CI 22.91, 114.07, I^2^ = 85%, REM) [38], [39], [52]. For the ten studies of 3 or 4 months, there was a relative benefit of 59.66 meters (95%CI 24.63, 94.69, I^2^ = 93%, REM) [37], [41], [44]–[46], [48], [49], [54], [55], [59]. When the three six-month studies were pooled, the benefit was 43.10 meters (95%CI 15.16, 71.04, I^2^ = 83%, REM) at EoT [36], [51], [53] (Table 3). However, the heterogeneity was high in each of these pools. When the study that was the most notable outlier in the funnel plot was excluded [59], the heterogeneity was reduced to 51% for the 3–4 month studies and the benefit was reduced to 46.45 meters (95%CI, 31.19, 61.71) but this was still clinically significant.

When studies were pooled according to type of RP, ten groups of studies were identified.

1. Salmeterol/Fluticasone plus CHM
  Two studies used salmeterol/fluticasone as RP, one for 6 months and one for 3 months [37], [53]. After 6 months there was a small non-significant increase in walking distance in the RP group of 5.06 meters. In the CHM plus RP group the increase of 28.27 meters was significant. At EoT there was a clinically significant difference between groups in favour of the test group (MD 25.27 m) [53]. In the three-month study, there was a significant improvement in both the RP and CHM plus RP groups. At EoT, the distance walked by those in the CHM plus RP group was significantly greater than in the group that only used salmeterol/fluticasone (MD 47.64 m) [37] (Table S5). The pooled data for this subgroup showed a clinically significant mean increase of 31.44 meters (95%CI 11.84, 51.04, I^2^ = 10%, REM) in the test groups compared to controls (Figure 5).
2. Theophylline (oral) plus pulmonary rehabilitation plus CHM
  In the study that used this RP for two months, there was a small non-significant increase in 6MWT in both the CHM plus RP group (MD 6.47 m) and the RP group (MD 2.63 m). There was no significant difference between groups at EoT [58] (Table S5).
3. Theophylline (oral) plus Mucosolvan (oral) plus Long-acting beta2-agonist (LABA) (inhaled) plus CHM
  Two studies used this RP for one month [39], [52]. In one study, the test group achieved a significantly greater distance over baseline of 118.00 meters. The RP group achieved a lower but significant increase (MD 60.00 meters). At EoT, there was a clinically significant benefit in favour of the CHM plus RP group of 52.00 meters [39]. In the other study, there was a significant improvement in both the CHM plus RP group (MD 112.00 m) and the RP group (MD 70.00 m) compared to baseline. After one month, the CHM plus RP group achieved greater mean walking distance (MD 38.00 m) compared with the RP group [52]. There was a clinically significant benefit for adding CHM in this subgroup (44.65 m, I^2^ = 0%, FEM) [39], [52] (Figure 5).
4. Aminophylline/Bromhexine/Chlorphenamine plus CHM
  In the study that used this compound oral drug for three months, there was a non-significant increase in 6MWT in the control group of 17.50 meters. A significant increase was observed in the test group of 72.30 meters and there was a clinically significant difference between groups of 57.60 meters at EoT (Table S5) [46].
5. Inhaled Ipratropium plus oxygen therapy plus CHM
  One study used inhaled ipratropium plus oxygen therapy as RP for one month. At EoT, the RP group remained relatively stable with a 6.00 meter increase but there was an improvement in the CHM plus RP group of 124.00 meters. At EoT, the addition of the CHM increased walking distance by 119 meters which was clinically significant (Table S5) [38].
6. Inhaled Ipratropium plus CHM
  One study used this RP for 6 months. The baseline mean 6MWD was almost the same in each group (T: 374 m, C: 378 m). After treatment, there was non-significant increase in walking distance in the control group of 23.00 meters, while the CHM plus ipratropium group produced a significant improvement of 62.00 meters. The relative increase of 35 meters between groups at the end of the 6 months was clinically significant (Table S5) [51].
7. Inhaled Ipratropium/Salbutamol plus oral Mucosolvan plus exercise plus CHM
  In one study, after six months treatment, walking distance significantly improved in both the control (MD 94.20 m) and CHM plus RP groups (MD 163.00 m). At EoT, the distance walked by those in the CHM plus RP group was greater by 65.08 meters than in the RP group which was clinically significant (Table S5) [36].
8. RP adjusted according to the guidelines plus CHM
  Five studies used RP adjusted for the individual according to the guidelines for three months [41], [45], [48], [49], [55]. In one study, the mean walking distance declined in the control group (MD −28.40 m) while it increased in the test group (MD 32.10 m) but neither change was significant. However, a clinically significant improvement of 55.30 meters was observed between groups at three months [48]. In another study, there was a shorter mean walking distance in the test group compared to control group at the beginning of the study (MD −37.88 m) but the difference was not significant. After treatment, both groups achieved non-significant improvements (C: 5.90 m; T: 28.82 m), so there was no significant difference between groups at EoT [49]. In the third study, improvement was non-significant in the control group (MD 22.10 m) but significant in the test group (MD 47.56 m) after treatment. At EoT the test group showed a relative increase in walking distance of 24.07 meters compared to control [41]. In the fourth study, the mean walking distance declined in the control group (MD −24.10 m) while it increased significantly in the test group (MD 33.20 m). A clinically significant improvement of 56.40 meters was observed between groups at three months [55]. In the remaining study, there was a significant increase in walking distance in the control group over baseline of 35.00 meters. In the test group, the addition of the CHM produced an improvement of 98.00 meters over baseline. At EoT, there was a clinically significant difference between groups in favour of CHM plus RP (MD 66.00 m) [45] (Table S5). The pooled data of this subgroup demonstrated a clinically significant improvement in 6MWT of 39.27 meters (95%CI 14.45, 64.09, I^2^ = 69%, REM) in favour of the CHM plus RP groups (Figure 5, Table 3) [41], [45], [48], [49], [55]. When the single study of patients who could walk more than 300 meters at baseline was removed [49], the result remained clinically significant at 48.40 meters (95%CI 28.11, 68.69) with heterogeneity reduced to 50%.
9. Unspecified RP plus CHM
  One study used CHM plus unspecified RP in the test group and showed clinically significant improvement within groups (C: 32.00 m; T: 87.00 m) as well as between groups (52.00 m, 95%CI 24.57, 79.43) after 4 months of treatment [44] (Table S5).
10. Unspecified RP with pulmonary rehabilitation plus CHM
  Two studies used CHM combined with unspecified RP plus p.r. in the test group [54], [59]. The greatest between-groups beneficial improvement was in the study that showed as an outlier in the funnel plot. The baseline mean walking distance was 375 meters in each group. After three months, a significant increase was observed in both the RP group (MD 75.34 m) and CHM plus RP group (MD 232.02 m). The test group showed a relative increase in walking distance of 156.02 meters compared to control at EoT [59]. In the other study, the baseline mean 6MWD was 20 meters further in the test group compared to control but this was not significant (Table S5). After three months the control showed a shorter mean walking distance of −21.40 meters whereas there was an improvement in the test group of 64.10 m but neither was significant. At EoT there was a significant benefit in favour of the CHM plus RP group of 105.50 meters [54]. The pooled effect at EoT was 153.65 meters in favour of the test groups which was significant with low heterogeneity (Figure 5, Table 3).

Overall, participants treated with CHM plus RP therapy achieved greater walking distances within 6 minutes compared to those treated with RP alone based on 17 studies (MD 54.61 m, 95%CI 33.30, 75.92, I^2^ = 91%, REM) but the heterogeneity was high [36]–[39], [41], [44]–[46], [48], [49], [51]–[55], [58], [59] (Figure 5). Therefore, sensitivity analysis was undertaken (Table 3). Analysis of data from the sixteen studies written in Chinese and from the 15 studies exclusively indexed in Chinese databases found similar results (Table 3).

When the three studies that used p.r. were removed [54], [58], [59], the total benefit of adding CHM was 48.86 meters (95%CI 36.07, 61.64, I^2^ = 71% REM). When the studies that used oxygen therapy [38], pulmonary exercise [36] and p.r. in addition to RP [54], [58], [59] were excluded from the meta-analysis, the mean difference decreased to 42.05 meters (95%CI 31.46, 52.63, REM) and I^2^ was reduced to 41%. When the studies with undefined RP [41], [44], [45], [48], [49], [55] were also excluded, the pooled benefit for the remaining six studies was 40.95 meters (95%CI 30.19, 52.89, I^2^ = 0%, FEM) which was clinically significant (Table 3).

When the seven studies of patients who could walk an average of 300 meters or more at baseline were considered separately [37], [39], [46], [49], [51]–[53], excluding two studies that used p.r. [58], [59], the benefit was reduced to 37.05 meters (Table 3). Conversely, for the 7 studies of patients who could walk less than 300 meters at baseline [36], [38], [41], [44], [45], [48], [55], excluding the one that used p.r. [54], the pooled benefit was greater (MD 60.73, Table 3).

In terms of the CHMs, twelve studies used formulae that contained *A. membranaceus*. There was a non-significant increase in 6MWT/D in the control groups of 22.96 meters. A significant increase was observed in the test groups of 80.86 meters and there was a significant difference between groups of 55.39 meters at EoT but heterogeneity was high [36]–[38], [41], [44], [45], [48], [49], [53], [55], [58], [59]. Removal of the outlier in the funnel plot reduced the benefit to 46.03 meters (95%CI 28.38, 63.67, I^2^ = 70%, REM) and removal of the four studies that used oxygen or p.r. as co-interventions [36], [38], [58], [59] reduced the benefit to 39.89 meters (95%CI 24.45, 55.33, I^2^ = 55% REM).

In the five studies that combined *A. membranaceus* with *A. macrocephala* the benefit for adding these CHMs was 49.56 meters (95%CI 32.08, 67.05, I^2^ = 60%, REM). When the study that also used p.r. [36] (Chen 12) was removed, the benefit remained clinically significant at 41.09 meters (95%CI 25.35, 56.84, I^2^ = 13%, REM).

For the five studies that contained cultured *C. sinensis* (CS) mycelia, the CHM plus RP groups achieved a significantly greater distance over baseline of 102.65 meters. The RP groups achieved a lower but significant increase of 35.56 meters. At EoT, there was a significant benefit in favour of the CS plus RP groups of 68.11 meters but heterogeneity was high [41], [45], [46], [51], [59]. When the outlier in the funnel plot [59] was removed the benefit was 44.56 meters (95%CI 25.37, 63.76, I^2^ = 54%, REM).

Two studies used the same formula with *Panax ginseng* as the main herb (Guo 2008 [39] and Zhang (1) 2007 [52]). The pooled effect for 6MWT was 44.65 meters in favour of the CHM plus RP groups after 1 month with no heterogeneity (Table 3).

### Adverse events

Seventeen adverse events were reported in 3 studies [37], [41], [43], with 7 adverse events in the CHM plus RP groups. Among these 7 cases, one had abdominal distension [43], one had thirst [43], one had palpitations [43], one had constipation [43], one had insomnia [43], and two had thirst and abdominal distension [41]. There was no significant difference between CHM plus RP and RP groups in rate of AEs (RR 0.53, 95%CI 0.15, 1.93, I^2^ = 0%). There was no adverse event reported in 6 studies [44], [50], [54]–[56], [58] and information on adverse events was not provided in 16 studies [36], [38]–[40], [42], [45]–[49], [51]–[53], [57], [59], [60].

## Discussion

This systematic review identified 25 randomized controlled trials of CHM plus RP versus RP for patients with stable COPD that included BODE Index and/or 6MWT/D as outcome measures. Meta-analysis found that compared with control groups that used RP, treatment that combined CHM plus the same RP produced a relative reduction in BODE Index in six out of seven studies. The pooled data showed a relative benefit of less than one point (−0.71) but four studies showed benefits of greater than one point [37], [40], [47], [54]. Consistent with the correlations found for the baseline and EoT scores, the benefits were more pronounced when the COPD was worse at baseline. In the three studies of patients with mean BODE Indices greater than 4, the benefit for adding CHM to RP was a clinically significant reduction of 1.40 points.

A systematic review has reported that the combination of CHM plus RP could increase SGRQ scores compared with the RPs alone [18]. Since the BODE Index was found to correlate well with SGRQ scores in a large cohort study with 10 years follow-up [31], the results of this review are consistent with the previous review.

There was a relative increase in the 6MWT/D in favour of CHM plus RP in 15 out of 17 studies. In the pooled data, the mean increase was 54.61 meters but there was considerable heterogeneity in the analysis. When the pool was reduced to six studies, there remained a clinically significant increase of 40.95 meters with no heterogeneity. A greater benefit for the addition of the CHMs was found when the walking distances were shorter at baseline. Overall, 14 of the 17 studies showed relative increases of 25 meters or more and would appear to have produced clinically important gains in exercise tolerance [24].

The combination of the CHMs and RPs appeared to be well-tolerated by patients. The AE incidence was balanced between the CHM plus RP and the RP groups, and none of the reports were serious events. However, AEs were reported by only 9 out of 25 studies so the safety data is incomplete.

### Limitations of the review

These promising observations should be interpreted with caution for several reasons. A range of methodological issues were identified. In particular, descriptions of the random sequence generation process and allocation concealment were lacking in the majority of studies. One study provided complete descriptions and used blind assessment but was not blind to patients and investigators [43]. It reported a significant benefit for 6MWT at EoT (6 months) and end of follow-up (18 months) but the data were presented as graphs and were not suitable for further analysis.

Overall, whether randomization and allocation concealment were effectively conducted in the majority of included studies remains unclear. Inadequate blinding may result in an overestimation of the effect in the test group. Only one study used an identical placebo to enable adequate blinding. This study found positive results for BODE Index and 6MWT in the combined CHM plus RP group, but the descriptions of randomisation and allocation concealment methods were not adequate and there was no assessment of the effectiveness of blinding, so the results may have been influenced by these methodological shortcomings [54].

The overemphasizing of positive effects arising from publication bias and language bias may be a limitation in this review. It includes only studies written in English or Chinese and all the included studies were conducted in China. Patients showed high compliance and may have expected a benefit from the addition of the CHM. This may have produced bias in patient performance or in measurement. Although the funnel plot for 6MWT/D showed a marked effect of the small studies, Egger's test was not suggestive of publication bias.

### Interpretation of the results

Although these studies used the same overall study design and the same outcome measures, the meaningfulness of pooled data is limited by a number of factors including the RPs used, study duration, the CHMs used and the severity of the COPD.

A range of different RPs was used and a number of studies treated individual participants differently according to their COPD severity as per the guidelines [34], [62]. Attempts were made to control for this source of variation by pooling studies that used the same specific RP.

For BODE Index, two studies used salmeterol/fluticasone (50 μg/500 μg) as RP [37], [60]. Participants in Chen 2009 [37] had higher baseline scores (6.31) than those in Yang 2013 (3.75) [60]. A benefit of −1.44 was observed after 3 months in Chen 2009 but there was only a 0.27 decrease after 1 month in Yang 2013 in the RP groups. This may suggest an effect of baseline difference in the BODE Index. In addition, the CHMs used in Chen 2009 were quite different from those in Yang 2013. Therefore, even though the pooled data showed no heterogeneity, the meaningfulness of the relative benefit of 0.70 for the addition of the CHMs is limited by clinical differences including treatment duration, the CHMs used and baseline scores.

For 6MWT, in the two studies that used salmeterol/fluticasone (50 μg/500 μg) [37], [53], Chen 2009 [37] found a benefit of 37.05 meters after 3 months but Zhang 2011 [53] found only a 5.06 meter increase after 6 months in the RP alone groups. These benefits were considerably lower than the 160 meter increase after 3 months using salmeterol/fluticasone (100 μg/250 μg) reported in a recent study from Iran [64]. However, the participants in each of these three studies had quite different exercise capacities at study commencement. In Mansori *et al* 2010 [64], the mean 6MWT at baseline was 300 meters, it was 327.42 meters in Chen 2009, and the patients in Zhang 2011 had the least impairment (385.42 m). These results suggest that greater benefits were evident in studies with lower exercise capacity at baseline. Consequently, even though the pooled benefit of 31.44 meters for this subgroup has low heterogeneity and the studies had the same duration, the patient groups were different.

For theophylline (oral) plus Mucosolvan (oral) plus LABA (inhaled), two studies were for one month [39], [52]. The mean walking distances were similar at baseline (386 m vs 378 m respectively) and the majority of participants were at COPD stage II in both studies. So these studies were directly comparable in terms of RP, duration and participants. In Guo 2008 [39] the RPs produced a 60.0 meter increase while in Zhang (1) 2007 [52] the increase was similar at 70.0 meters. The pooled effect was 65.36 meters (95%CI: 45.17, 85.55, I^2^ = 0%). In this sub-group the relative benefit found in the pooled data of 44.65 meters (I^2^ = 0%) for the addition of the CHM is based on closely comparable studies.

In terms of the CHMs used in the above four studies, both Guo 2008 [39] and Zhang (1) 2007 [52] used the same CHM formula with the only difference being that Guo 2008 used a capsule while Zhang (1) 2007 used a decoction. Both studies produced clinically meaningful improvements in exercise tolerance. This strengthens the likelihood that the addition of this CHM to theophylline (oral) plus Mucosolvan (oral) plus LABA (inhaled) had real effects, over the short term at least. For the salmeterol/fluicasone group, Chen 2009 [37] and Zhang 2011 [53] used CHMs that showed distinct similarities in terms of their ingredients. Chen 2009 showed the greater benefit but due to the differences at baseline and the different study durations, it was not possible to determine whether the CHM used for 3 months in Chen 2009 was more effective than that used for 6 months in Zhang 2011.

Studies were also pooled when the RP was similar in that it was administered according to the COPD severity in accord with the GOLD or the CSRD guidelines. For BODE Index, there was no benefit for Xu (1) 2012 and a significant improvement in Yu 2011 but the pool produced a non-significant result with high heterogeneity (I^2^ = 74%). Both studies were for 3 months but RP was according to CSRD in Xu (1) 2012 and according to GOLD in Yu 2011. Xu (1) 2012 used two different CHMs according to syndrome differentiation and both these CHMs were quite different to the CHM used Yu 2011. This limits the meaningfulness of the pool.

For 6MWT, the pool included five studies each with durations of 3 months [41], [45], [48], [49], [55]. The pooled data showed a significant relative improvement of 39.27 meters but the heterogeneity was relatively high (I^2^ = 69%). Xu (1) 2012 [49] had a considerably longer mean walking distance at baseline (>411 m) than the other four studies (248 m–294 m) and this study was the major contributor to the heterogeneity. When removed, the pooled relative benefit was 48.0 meters (I^2^ = 50%). Within the remaining four studies, the greatest relative benefit was in Liu 2009 [45] (66.00 m). This study only included participants who conformed to the indications of the CHMs used, which may have produced a more targeted effect. However, it is difficult to compare the effects of this CHM with those used in the other three studies since Xu (2) 2012 [48] and Fan 2013 [55] included participants with different TCM syndromes while Huang 2005 [41] did not specify whether TCM syndrome differentiation was used in selection.

For the results of data pooling to be fully interpretable, studies of CHM plus RP need to be comparable in terms of not only the RP used and duration, but also the CHMs used and the appropriateness of the CHMs to the participant population. With regard to this aspect, thirteen of the 25 studies included TCM syndrome differentiation in participant selection. Of these, Li 2012 [43] and Xu (1) 2012 [49] allowed multiple syndromes and CHM selection according to syndrome. This more closely reflects actual practice but would appear to introduce additional sources of variation into a clinical trial. In the case of Xu (1) 2012, [49] large SDs were apparent for 6MWT and no significant differences were found over baseline for test or control groups at three months. Li 2012 [43] reported no significant benefit for the CHM at 3 months but found a benefit at 6 months of treatment which was still evident one year post-trial. However this study did not report data suitable for inclusion in the meta-analyses.

### Effect of baseline values on outcomes

Significant negative correlations between baseline values and change at EoT were found for BODE Index and walking distance in both test and control groups. For 6MWT/D, the correlation found in the test groups was stronger than that in the control groups since there was decline in exercise tolerance in some studies and no change in others. Similarly, the correlation found in the test groups was twice that in the control groups for the BODE Index. In the case of the RP groups, in most studies it is not clear whether the participants had been using the same RP prior to the study or whether the RP was a new intervention. In the first case, it could be expected that most of the benefit for the RP would have been achieved prior to the trial and scores would not show much change during the trial, so this may account for some of the variability in the intervention effect found in the RP groups. Whether this effect holds for all types of interventions has not been further explored, but these correlations suggest that when BODE Index and 6MWT are used as outcome measures in clinical trials, the baseline values need to be considered when undertaking sample size calculations and when interpreting results.

### Effects of the main herbs and main formulae

A previous review found that *Astragalus membranaceus* (AM) was the most frequently used herb in trials of CHM for COPD [18]. Similarly, it was frequently used in the studies that used BODE Index (n = 5) and 6MWT/D (n = 13) as outcomes. Significant benefits were found in the pooled data for studies of CHMs containing AM, with a decrease in the BODE Index of 0.62 points [37], [40], [49], [58], [60] and an increase in walking distance of 49.27 meters (Table 3) [36]–[38], [41], [44], [48], [49], [53], [55], [58], [59] for the test groups compared to the RP controls. This effect is supported by a recent review that found that oral Huangqi formulae had beneficial effects on lung function, quality of life, COPD symptoms, and incidence of exacerbations for patients with stable COPD [65].

The two studies that used *Panax ginseng* as the main herb showed a clinically significant 6MWD benefit of 44.65 meters in favour of the CHM plus RP groups [39], [52]. This enhanced exercise tolerance is consistent with two recent reviews. One mentioned that *P. ginseng* may improve respiratory muscle strength and lung function [66]. Another review of ginseng-containing herbal formulae reported improvements in respiratory volume and quality of life [67].

The most commonly used formula (n = 5) contained cultured *Cordyceps sinensis* mycelia alone under the names ‘Bailing Capsule’ or ‘Jinshuibao Capsule’. *C. sinensis* (CS) is a kind of fungus that is parasitic on the larvae of *Lepidoptera* species. It has been widely used as a time-honoured tonic food and herbal medicine in China [68]. Its production is so limited that it cannot be widely used and a cultured form is used instead. For the five studies that reported 6MWT, there was a relative benefit of 44.56 meters in the pooled data (Table 3) [41], [45], [46], [51]. The one study in the BODE Index group reported a relative benefit of −1.21 points [40] in favour of the CS plus RP groups.

AM and CS are thought to strengthen the body's resistance to illness through effects on the immune system. Experimental studies have found that Astragalus polysaccharides (APS) extracted from AM had immunomodulatory activity by activating macrophages [14]. CS has been reported to have immunomodulatory [11]–[13] and anti-inflammatory activity [7]–[9]. In addition, effects on exercise have been investigated for these two herbs. Rats that received *A. membranaceus* flavonoids (AMF) showed higher endurance capacity in a swimming test when compared with those without AMF administration [69]. Also, CS supplementation, with or without exercise, was reported to improve exercise endurance capacity in rats by activating the skeletal muscle metabolic regulators [70].

The experimental studies suggest that each of the principal herbs, *A. membranaceus, P. ginseng* and *C. sinensis*, can benefit resistance to illness and improve exercise tolerance but it remains unclear whether these herbs are appropriate for all patients and whether particular combinations of herbs have additive effects in a clinical setting.

## Conclusions

CHM appears to be a well-tolerated addition to routine pharmacotherapy for stable COPD. The meta-analysis results indicate that the addition of CHM to routine pharmacotherapies may produce additional benefits in terms of decreasing the BODE Index and increasing the 6MWD in stable COPD patients when used for up to six months. These potential benefits of CHM as a supportive intervention need to be further evaluated through trials that address the identified methodological deficiencies, have published protocols, provide quality-control data for both the CHM and control interventions and are of sufficient duration to assess any change. Trials should employ an identical placebo for the CHM in the control group to enable effective blinding. In order for outcomes to be fully interpretable, participants should have similar COPD severity at baseline, the PR should be standardised and the trial should investigate a single CHM intervention that is appropriate to the participant group.

## Supporting Information

Figure S1 **Funnel plot of 17 studies evaluating the effect of CHM plus RP for stable COPD on 6MWT/D.** MD: mean difference, SE: standard error.
(TIF)

Table S1 **Search terms used in PubMed.** MeSH: MeSH terms, TW: text word.
(DOCX)

Table S2 **Characteristics of the 25 studies of CHM plus RP for stable COPD.** CHM: Chinese Herbal Medicine, RP: routine pharmacotherapy, mths: months, R/A: registration/analysis, M/F: male/female, yrs: years, CM: Chinese Medicine, NS: not stated, T: test group, C: control group, CVD: cerebrovascular disease. AE: acute exacerbation.
(DOCX)

Table S3 **Interventions of the 25 studies of CHM plus RP for stable COPD.** CHM: Chinese Herbal Medicine, RP: routine pharmacotherapy, Y: yes, N: no, CSRD: COPD Study Group of Chinese Society of Respiratory Disease: Treatment guidelines of COPD, GOLD: Global Initiative for Chronic Obstructive Lung Disease.
(DOCX)

Table S4 **Results for individual studies: BODE Index (MD, 95%CI) for the CHM plus RP groups (T) and the RP groups (C) at baseline and end of treatment.** *Significant difference, MD: mean difference, CI: confidence interval, CHM: Chinese Herbal Medicine, RP: routine pharmacotherapy, T: test group, C: control group, EoT: end of treatment, mths: months, Ipra.: ipratropium (inhaled), Salb.: salbutamol (inhaled), Theo.(+p.r.): oral theophylline (plus pulmonary rehabilitation), Theo.: theophylline (oral), Salm./Flu.: salmeterol/fluticasone (inhaled), RP (guidelines): routine pharmacotherapy (adjusted for severity according to guidelines), RP(+ p.r.): routine pharmacotherapy (pharmacotherapy plus pulmonary rehabilitation).
(DOCX)

Table S5 **Results for individual studies: 6MWT/D (MD, 95%CI, meters) for the CHM plus RP groups (T) and the RP groups (C) at baseline and end of treatment.** *Significant difference, CHM: Chinese Herbal Medicine, RP: routine pharmacotherapy, MD: mean difference, mths: months, CI: confidence interval, T: test group, C: control group, EoT: end of treatment, Salm./Flu.: salmeterol/fluticasone (inhaled), Theo.(+p.r.): oral theophylline (plus pulmonary rehabilitation), Theo.: theophylline (oral), Muc.: Mucosolvan (oral), LABA: long-acting β2 agonist (inhaled), Ami./Bro./Chlo.: compound oral medication which contained aminophylline, bromhexine and chlorphenamine, Ipra.: ipratropium (inhaled), oxy.: oxygen therapy, Salb./Ipra.: salbutamol/ipratropium (inhaled), exe.: pulmonary exercise, RP(guidelines): routine pharmacotherapy (adjusted for severity according to guidelines), RP(+ p.r.): routine pharmacotherapy (pharmacotherapy plus pulmonary rehabilitation).
(DOCX)

Checklist S1 **PRISMA checklist.**
(DOC)

## References

[1] Qaseem A, Wilt TJ, Weinberger SE, Hanania NA, Criner G, et al (2011) Diagnosis and management of stable chronic obstructive pulmonary disease: a clinical practice guideline update from the American College of Physicians, American College of Chest Physicians, American Thoracic Society, and European Respiratory Society. Ann Intern Med 155: 179–191. 21810710 10.7326/0003-4819-155-3-201108020-00008

[2] Murray CJ, Lopez AD (1997) Alternative projections of mortality and disability by cause 1990-2020: Global Burden of Disease Study. Lancet 349: 1498–1504. 9167458 10.1016/S0140-6736(96)07492-2

[3] George J, Ioannides-Demos LL, Santamaria NM, Kong DC, Stewart K (2004) Use of complementary and alternative medicines by patients with chronic obstructive pulmonary disease. Med J Aust 181: 248–251. 15347271 10.5694/j.1326-5377.2004.tb06262.x

[4] Hu J, Zhang J, Zhao W, Zhang Y, Zhang L, et al (2011) Cochrane systematic reviews of Chinese herbal medicines: an overview. PLoS One 6: e28696. 22174870 10.1371/journal.pone.0028696 PMC3235143

[5] Vickers A, Zollman C (1999) ABC of complementary medicine: herbal medicine. BMJ 319: 1050–1053. 10521203 10.1136/bmj.319.7216.1050 PMC1116847

[6] Shin IS, Lee MY, Lim HS, Ha H, Seo CS, et al (2012) An extract of Crataegus pinnatifida fruit attenuates airway inflammation by modulation of matrix metalloproteinase-9 in ovalbumin induced asthma. PLoS One 7: e45734. 23029210 10.1371/journal.pone.0045734 PMC3448716

[7] Kim HG, Shrestha B, Lim SY, Yoon DH, Chang WC, et al (2006) Cordycepin inhibits lipopolysaccharide-induced inflammation by the suppression of NF-kappaB through Akt and p38 inhibition in RAW 264.7 macrophage cells. Eur J Pharmacol 545: 192–199. 16899239 10.1016/j.ejphar.2006.06.047

[8] Rao YK, Fang SH, Tzeng YM (2007) Evaluation of the anti-inflammatory and anti-proliferation tumoral cells activities of Antrodia camphorata, Cordyceps sinensis, and Cinnamomum osmophloeum bark extracts. J Ethnopharmacol 114: 78–85. 17822865 10.1016/j.jep.2007.07.028

[9] Shahed AR, Kim SI, Shoskes DA (2001) Down-regulation of apoptotic and inflammatory genes by Cordyceps sinensis extract in rat kidney following ischemia/reperfusion. Transplant Proc 33: 2986–2987. 11543822 10.1016/s0041-1345(01)02282-5

[10] Denzler KL, Waters R, Jacobs BL, Rochon Y, Langland JO (2010) Regulation of inflammatory gene expression in PBMCs by immunostimulatory botanicals. PLoS One 5: e12561. 20838436 10.1371/journal.pone.0012561 PMC2933230

[11] Koh JH, Yu KW, Suh HJ, Choi YM, Ahn TS (2002) Activation of macrophages and the intestinal immune system by an orally administered decoction from cultured mycelia of Cordyceps sinensis. Biosci Biotechnol Biochem 66: 407–411. 11999417 10.1271/bbb.66.407

[12] Kuo YC, Tsai WJ, Shiao MS, Chen CF, Lin CY (1996) Cordyceps sinensis as an immunomodulatory agent. Am J Chin Med 24: 111–125. 8874668 10.1142/S0192415X96000165

[13] Kuo YC, Tsai WJ, Wang JY, Chang SC, Lin CY, et al (2001) Regulation of bronchoalveolar lavage fluids cell function by the immunomodulatory agents from Cordyceps sinensis. Life Sci 68: 1067–1082. 11212870 10.1016/s0024-3205(00)01011-0

[14] Shao BM, Xu W, Dai H, Tu P, Li Z, et al (2004) A study on the immune receptors for polysaccharides from the roots of Astragalus membranaceus, a Chinese medicinal herb. Biochem Biophys Res Commun 320: 1103–1111. 15249203 10.1016/j.bbrc.2004.06.065

[15] Chen N, Li H, Zhao LY, Liu JB, Huang Q, et al (2009) [Therapy for clearing heat and resolving phlegm in treatment of systemic inflammatory response syndrome in acute deterioration stage of chronic obstructive pulmonary disease: a randomized controlled trial]. Zhong Xi Yi Jie He Xue Bao 7: 105–109. 19216850 10.3736/jcim20090202

[16] Cui P, Wang YN, Gao TS, Qi TC, Mei L, et al (2012) [Study on mechanism of traditional Chinese medicines reducing phlegm and resolving masses in treatment of goiter]. Zhongguo Zhong Yao Za Zhi 37: 3451–3456. 23373220

[17] Chan E, Tan M, Xin J, Sudarsanam S, Johnson DE (2010) Interactions between traditional Chinese medicines and Western therapeutics. Curr Opin Drug Discov Devel 13: 50–65. 20047146

[18] An X, Zhang AL, May BH, Lin L, Xu Y, et al (2012) Oral Chinese herbal medicine for improvement of quality of life in patients with stable chronic obstructive pulmonary disease: a systematic review. J Altern Complement Med 18: 731–743. 22803654 10.1089/acm.2011.0389 PMC3421964

[19] Jones PW (2001) Health status measurement in chronic obstructive pulmonary disease. Thorax 56: 880–887. 11641515 10.1136/thorax.56.11.880 PMC1745959

[20] Glaab T, Vogelmeier C, Buhl R (2010) Outcome measures in chronic obstructive pulmonary disease (COPD): strengths and limitations. Respir Res 11: 79. 20565728 10.1186/1465-9921-11-79 PMC2902430

[21] Pinto-Plata VM, Cote C, Cabral H, Taylor J, Celli BR (2004) The 6-min walk distance: change over time and value as a predictor of survival in severe COPD. Eur Respir J 23: 28–33. 14738227 10.1183/09031936.03.00034603

[22] Brown CD, Wise RA (2007) Field tests of exercise in COPD: the six-minute walk test and the shuttle walk test. COPD 4: 217–223. 17729065 10.1080/15412550701480125

[23] Wise RA, Brown CD (2005) Minimal clinically important differences in the six-minute walk test and the incremental shuttle walking test. COPD 2: 125–129. 17136972 10.1081/copd-200050527

[24] Holland AE, Hill CJ, Rasekaba T, Lee A, Naughton MT, et al (2010) Updating the minimal important difference for six-minute walk distance in patients with chronic obstructive pulmonary disease. Arch Phys Med Rehabil 91: 221–225. 20159125 10.1016/j.apmr.2009.10.017

[25] Spruit MA, Polkey MI, Celli B, Edwards LD, Watkins ML, et al (2012) Predicting outcomes from 6-minute walk distance in chronic obstructive pulmonary disease. Joural of the American Medical Directors Association 13: 291–297. 10.1016/j.jamda.2011.06.009 21778120

[26] Cote CG, Casanova C, Marin JM, Lopez MV, Pinto-Plata V, et al (2008) Validation and comparison of reference equations for the 6-min walk distance test. Eur Respir J 31: 571–578. 17989117 10.1183/09031936.00104507

[27] Celli BR, Cote CG, Marin JM, Casanova C, Montes de Oca M, et al (2004) The body-mass index, airflow obstruction, dyspnea, and exercise capacity index in chronic obstructive pulmonary disease. N Engl J Med 350: 1005–1012. 14999112 10.1056/NEJMoa021322

[28] Ong KC, Earnest A, Lu SJ (2005) A multidimensional grading system (BODE index) as predictor of hospitalization for COPD. Chest 128: 3810–3816. 16354849 10.1378/chest.128.6.3810

[29] Pinto-Plata V, Casanova C, Mullerova H, de Torres JP, Corado H, et al (2012) Inflammatory and repair serum biomarker pattern. Association to clinical outcomes in COPD. Respir Res 13: 71. 22906131 10.1186/1465-9921-13-71 PMC3493287

[30] Faganello MM, Tanni SE, Sanchez FF, Pelegrino NR, Lucheta PA, et al (2010) BODE index and GOLD staging as predictors of 1-year exacerbation risk in chronic obstructive pulmonary disease. Am J Med Sci 339: 10–14. 19926966 10.1097/MAJ.0b013e3181bb8111

[31] Marin JM, Cote CG, Diaz O, Lisboa C, Casanova C, et al (2011) Prognostic assessment in COPD: health related quality of life and the BODE index. Respir Med 105: 916–921. 21282050 10.1016/j.rmed.2011.01.007

[32] Imfeld S, Bloch KE, Weder W, Russi EW (2006) The BODE index after lung volume reduction surgery correlates with survival. Chest 129: 873–878. 16608932 10.1378/chest.129.4.873

[33] Shang Q, Xu H, Huang L (2012) Tanshinone IIA: A Promising Natural Cardioprotective Agent. Evid Based Complement Alternat Med 2012: 716459. 22454677 10.1155/2012/716459 PMC3292221

[34] GOLD (2011) Global Strategy for Diagnosis, Management and Prevention of COPD The Global Initiative for Chronic Obstructive Lung Disease (GOLD).

[35] Higgins JPT, Green S, eds (2011) Cochrane Handbook for Systematic Reviews of Interventions Version 5.1.0. The Cochrane Collaboration.

[36] Chen GC, Li YX, Zhou ZY, Xue HR, Zhang YB (2012) Combination of Buzhong-Yiqi decoction and routine treatment for stable chronic obstructive pulmonary disease [in Chinese]. Shanghai Journal of Traditional Chinese Medicine 46: 42–44.

[37] Chen Q, Hong XC, Cai Y, Ye L (2009) Clinical observation of integrated Chinese and western medicine treatment in patients with stable COPD [in Chinese]. Journal of Fujian University of Traditional Chinese Medicine 19: 12–14.

[38] Cui CB, Yuan YD, Liu SH, Han DL, Gao XL, et al (2004) Intervention effect of Tongfei mixture on nocturnal hypoxia in patients with chronic obstructive pulmonary disease [in Chinese]. Chinese Journal of Integrated Traditional and Western Medicine 24: 885–888. 15553819

[39] Guo WX, Zhang FY, Yan GL (2008) Clinical observation of the therapeutic effect of Jianfei capsule on chronic obstructive pulmonary disease at stationary phase [in Chinese]. Hebei Journal of Traditional Chinese Medicine 30: 121–125.

[40] Hu QG, Liu LL (2012) Clinical observation of Jinshuibao capsule combined with Bufeihuoxue capsule in treating patients with stable COPD [in Chinese]. Journal of Guiyang Collage of Traditional Chinese Medicine 34: 135–137.

[41] Huang DH, Wu L, He DP, Lin L (2005) Clinical observation of tranquilization period of COPD treated by integration of traditional Chinese medicine and western medicine [in Chinese]. Journal of the Fourth Military Medical University 26: 1611–1613.

[42] Jian XY, Huang SQ, Lai X, Chen WY, Jiang RB (2012) Influence of Pingchuan capsules on BODE indexes in patients with stable chronic obstructive pulmonary disease [in Chinese]. Journal of Beijing University of Traditional Chinese Medicine (TCM clinical edition) 19: 32–34.

[43] Li SY, Li JS, Wang MH, Xie Y, Yu XQ, et al (2012) Effects of comprehensive therapy based on traditional Chinese medicine patterns in stable chronic obstructive pulmonary disease: a four-center, open-label, randomized, controlled study. BMC Complement Altern Med 12: 197. 23107470 10.1186/1472-6882-12-197 PMC3528455

[44] Liao YL, An W, Zhang W (2011) Stable COPD patients treated with Yupingfengsan in addition to routine therapy: 30 cases [in Chinese]. Guiding Journal of Traditional Chinese Medicine and Pharmacy 30: 63–66.

[45] Liu CS (2009) Clinical observation on the treatment of 30 cases of chronic obstructive pulmonary disease at stable term by benefiting vital energy and promoting blood, resolving phlegm and dredging collaterals [in Chinese]. Guiding Journal of Traditional Chinese Medicine and Pharmacy 11: 10–12.

[46] Mao R (2009) Clinical observation of Bailing capsules in treating stable COPD [in Chinese]. Chinese Journal of Integrated Traditional and Western Medicine 29: 362–367.

[47] Shan LN, Liu XH, Zhong LH (2011) The effects of integrated Chinese and western medicine on prognostic indexes of patients with stable chronic obstructive pulmonary disease [in Chinese]. Journal of Guangzhou University of Traditional Chinese Medicine 28: 590–592.

[48] Xu JZ, Fan DB, Qin XP, Yang L, Bai HH, et al (2012) Clinical observation of Gujingao in the treatment of 45 participants with stable COPD [in Chinese]. Yunnan Journal of Traditional Chinese Medicine and Materia Medica 33: 31–32.

[49] Xu TZ, Yao XL (2012) Effect observation on treating stable stage of COPD with therapy of Winter Disease In Summer [in Chinese]. Chinese archives of Traditional Chinese Medicine 30: 570–572.

[50] Yu JY (2011) The effects of Gejiedingchuanjiaonang on BODE index in patients with stable chronic obstructive pulmonary disease [in Chinese]. Journal of Medical Information 4019.

[51] Zhang FY, Liu YH, Li SF (2007) Effects of Bailing capsule combined with ipratropium bromide aerosol on the lung funtion and quality of life in patients with stable COPD [in Chinese]. Journal of Emergency in Traditional Chinese Medicine 19: 198–209.

[52] Zhang FY, Wang YL, Li SF (2007) The effects of Basic Chinese Herbal Formula combined with conventional therapy for COPD patients and their exercise tolerence [in Chinese]. Shanxi Zhongyi Journal 28: 1594–1596.

[53] Zhang RZ, Zheng SJ, Yan GZ (2011) Observation of the effects of Yupingfengsan combined with Jinshuiliujunjian treatment in patients with modrate COPD [in Chinese]. China Practical Medicine 6: 118–119.

[54] Zhao YL, Song HR, Fei JX, Liang Y, Zhang BH, et al (2012) The effects of Chinese Yam-Epimedium mixture on respiratory function and quality of life in patients with chronic obstructive pulmonary disease. Journal of Traditional Chinese Medicine 32: 203–207. 22876444 10.1016/s0254-6272(13)60012-6

[55] Fan BD, Qin XP, Xu JZ, Bai HH, Yu KS, et al.. (2012) Clinical observation on the treatment of Bu-Fei-Jian-Pi-Yi-Shen decoction combined with western medicine for 90 patients with stable COPD [in Chinese]. Journal of Sichuan of Traditional Chinese Medicine: 83–85.

[56] Liang AW, Tan YP, Liang W, Nong TQ, Su QJ, et al.. (2013) The effect of Run-Fei-Jian-Pi-Bu-Shen decoction on improvement of symptoms and 6MWT of patients with stable COPD [in Chinese]. Journal of Emergency in Traditional Chinese Medicine: 1125–1127.

[57] Lin YZ (2013) Clinical observation on integrated Chinese and western medicine treating stable COPD [in Chinese]. Chinese Manipulation and Rehabilitation Medicine: 103–105.

[58] Peng ZQ (2013) The effect of No. 1-Bu-Fei decoction on patients with stable COPD of Lung Qi Deficiency syndrome and influence of MMP-9 [Master degree thesis in Chinese]: Fujian University of Traditional Chinese Medicine.

[59] Zeng JQ, Liao ZC, Wang SM, Lin LS, Chen WY, et al.. (2013) Clinical observation of modified Si-Jun-Zi granules in improving exercise tolerance of patients with COPD [in Chinese]. Journal of New Chinese Medicine: 32–34.

[60] Yang CM, Lu YW, He YH (2013) Clinical observation of Tong-Qi-Pai-Yong decoction combined with Seretide for stable COPD [in Chinese]. Modern Journal of Integrated Traditional Chinese and Western Medicine: 729–730.

[61] Society AT (1999) Pulmonary rehabilitation-1999. Am J Respir Crit Care Med 159: 1666–1682. 10228143 10.1164/ajrccm.159.5.ats2-99

[62] CSRD (2007) Chinese Society of Respiratory Disease (CSRD): guideline for diagnosis and management of chronic obstructive pulmonary disease (revision 2007). Chin J Inrern Med 46: 254–261.

[63] Cote CG, Celli BR (2005) Pulmonary rehabilitation and the BODE index in COPD. Eur Respir J 26: 630–636. 16204593 10.1183/09031936.05.00045505

[64] Mansori F, Nemat Khorasani A, Boskabady MH, Boskabady M (2010) The effect of inhaled salmeterol, alone and in combination with fluticasone propionate, on management of COPD patients. Clin Respir J 4: 241–247. 20887348 10.1111/j.1752-699X.2010.00185.x

[65] Wu L, Chen YB, Xu YJ, Guo XF, Li XY, et al (2013) Oral huangqi formulae for stable chronic obstructive pulmonary disease: a systematic review and meta-analysis. Evid Based Complement Alternat Med 2013: 705315. 23606889 10.1155/2013/705315 PMC3623121

[66] Shergis JL, Zhang AL, Zhou W, Xue CC (2013) Panax ginseng in Randomised Controlled Trials: A Systematic Review. Phytother Res 27: 949–965. 22969004 10.1002/ptr.4832

[67] An X, Zhang AL, Yang AW, Lin L, Wu D, et al (2011) Oral ginseng formulae for stable chronic obstructive pulmonary disease: a systematic review. Respir Med 105: 165–176. 21146973 10.1016/j.rmed.2010.11.007

[68] Li CY, Chiang CS, Cheng WC, Wang SC, Cheng HT, et al (2012) Gene expression profiling of dendritic cells in different physiological stages under Cordyceps sinensis treatment. PLoS One 7: e40824. 22829888 10.1371/journal.pone.0040824 PMC3400664

[69] Kuo YH, Tsai WJ, Loke SH, Wu TS, Chiou WF (2009) Astragalus membranaceus flavonoids (AMF) ameliorate chronic fatigue syndrome induced by food intake restriction plus forced swimming. J Ethnopharmacol 122: 28–34. 19103273 10.1016/j.jep.2008.11.025

[70] Kumar R, Negi PS, Singh B, Ilavazhagan G, Bhargava K, et al (2011) Cordyceps sinensis promotes exercise endurance capacity of rats by activating skeletal muscle metabolic regulators. J Ethnopharmacol 136: 260–266. 21549819 10.1016/j.jep.2011.04.040

