# Supplementary figures and images for: Oral Chinese Herbal Medicine Combined with Pharmacotherapy for Stable COPD: A Systematic Review of Effect on BODE Index and Six Minute Walk Test
Source: PLoS One. 2014 Mar 12;9(3):e91830. doi: 10.1371/journal.pone.0091830 (PMC3951501; doi:10.1371/journal.pone.0091830)

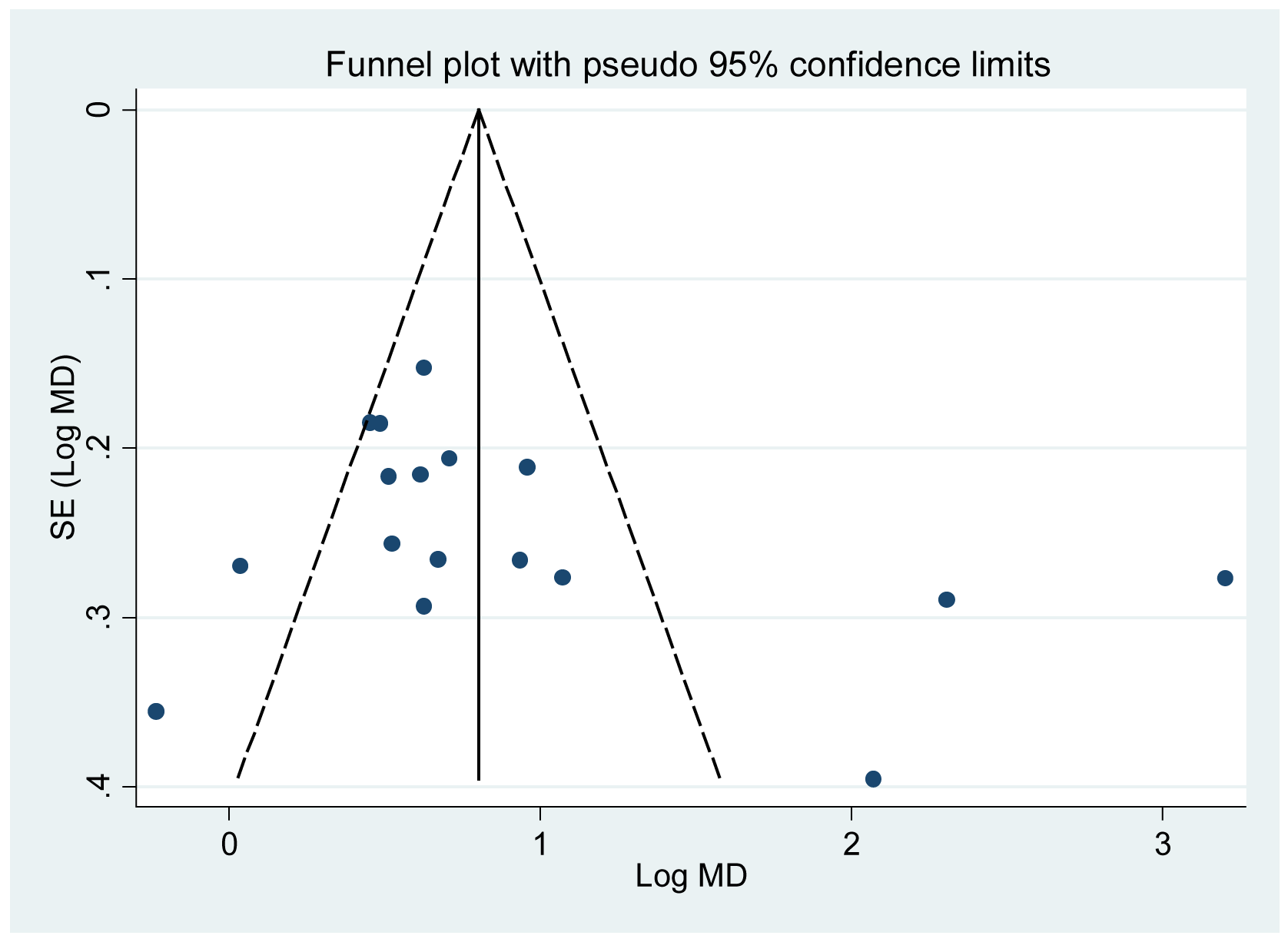

Supplement: Figure S1 — Funnel plot of 17 studies evaluating the effect of CHM plus RP for stable COPD on 6MWT/D. MD: mean difference, SE: standard error. (TIF) [file pone.0091830.s001.tif]
