# Supplementary material for: Oral Chinese Herbal Medicine Combined with Pharmacotherapy for Stable COPD: A Systematic Review of Effect on BODE Index and Six Minute Walk Test
Source: PLoS One. 2014 Mar 12;9(3):e91830. doi: 10.1371/journal.pone.0091830 (PMC3951501; doi:10.1371/journal.pone.0091830)
Supplement: Table S1 — Search terms used in PubMed. MeSH: MeSH terms, TW: text word. (DOCX) [file pone.0091830.s002.docx]

# Table S1 Search terms used in PubMed

|  | Search terms |
| --- | --- |
| condition | 1. Pulmonary Disease, Chronic Obstructive [MeSH] 2. Bronchitis, Chronic [MeSH] 3. Pulmonary Emphysema [MeSH] 4. Emphysema [MeSH] 5. COPD [TW] 6. Chronic Obstructive Pulmonary [TW] 7. COAD [TW] 8. AECB [TW] 9. COBD [TW] 10. Chronic Obstructive Airway [TW] 11. Chronic Obstructive Lung [TW] 12. Chronic obstructive bronchopulmonary [TW] 13. Chronic obstructive respiratory [TW] 14. Chronic Airflow Obstruction [TW] 15. Chronic Airflow Obstructive [TW] 16. Chronic bronchitis [TW] 17. Pulmonary emphysema [TW] 18. Lung emphysema [TW] 19. Chronic Airflow limitation [TW] 20. #1 - #19/OR |
| intervention | 1. Medicine, Chinese Traditional [MeSH] 2. Phytotherapy [MeSH] 3. Plant Extracts [MeSH] 4. Plants, Medicinal [MeSH] 5. Materia Medica [MeSH] 6. Complementary therap* [TW] 7. Complementary Medicine* [TW] 8. Alternative Medicine* [TW] 9. Alternative therap* [TW] 10. Traditional Medicine* [TW] 11. Ethnomedicine* [TW] 12. Ethnopharmacology [TW] 13. Ethnobotany [TW] 14. Oriental Traditional Medicine*[TW] 15. Oriental Medicine* [TW] 16. Traditional Chinese Medicine*[TW] 17. TCM [TW] 18. Chinese Medicine* [TW] 19. Kanpo [TW] 20. Kampo [TW] 21. Phytotherap* [TW] 22. Plant Extract* [TW] 23. Plant Drug* [TW] 24. Herbal Drug* [TW] 25. Medicinal Plant* [TW] 26. Pharmaceutical Plant* [TW] 27. Medicinal Herb* [TW] 28. Materia Medica* [TW] 29. Herb [TW] OR Herbs [TW] 30. Herbal Medicine* [TW] 31. Chinese Medicinal [TW] 32. Herbology [TW] 33. #21 - #52/OR |
| study type | 1. Clinical Trial [Publication Type] 2. "Clinical Trials as Topic" [Mesh] 3. "Comparative Study" [Publication Type] 4. "Evaluation Studies" [Publication Type] 5. "Evaluation Studies as Topic"[Mesh] 6. "Case Reports" [Publication Type] 7. Case-Control Studies [Mesh] 8. Cohort Studies [Mesh] 9. Cross-Sectional Studies [Mesh] 10. "Cross-Over Studies" [Mesh] 11. Observation [Mesh] 12. Control Groups [Mesh] 13. Random Allocation [Mesh] 14. Double-Blind Method [Mesh] 15. Single-blind method [Mesh] 16. Double-Blind [TW] 17. Single-blind [TW] 18. Blinding [TW] 19. Double-Masked [TW] 20. Single-Masked [TW] 21. #54 - #73/OR |
|  | 1. #20 AND #53 AND #74 |
