# Supplementary material for: Oral Chinese Herbal Medicine Combined with Pharmacotherapy for Stable COPD: A Systematic Review of Effect on BODE Index and Six Minute Walk Test
Source: PLoS One. 2014 Mar 12;9(3):e91830. doi: 10.1371/journal.pone.0091830 (PMC3951501; doi:10.1371/journal.pone.0091830)
Supplement: Table S2 — Characteristics of the 25 studies of CHM plus RP for stable COPD. CHM: Chinese Herbal Medicine, RP: routine pharmacotherapy, mths: months, R/A: registration/analysis, M/F: male/female, yrs: years, CM: Chinese Medicine, NS: not stated, T: test group, C: control group, CVD: cerebrovascular disease. AE: acute exacerbation. (DOCX) [file pone.0091830.s003.docx]

**Table S2 Characteristics of the 25 studies of CHM plus RP for stable COPD**

| First author, year | Location (duration/follow-up: mths) | Sample size/Dropout(reasons) | No. patients (R/A) | M/F | Age: Mean±SD (Range) | Severity/COPD history (yrs) | CM syndrome differentiation |
| --- | --- | --- | --- | --- | --- | --- | --- |
| Chen, 2009 [37] | China (3/0) | 60/NS | T: 30/30, C: 30/30 | total: 29/31 | total: 70.1±NS | NS/NS | NS |
| Chen, 2012 [36] | China (6/0) | 80/0 | T: 40/40, C: 40/40 | T: 29/11, C:31/9 | T: 62.20±12.67, C: 61.23±11.50 | NS/T: 12.50±4.88, C: 14.12±5.3 | NS |
| Cui, 2004 [38] | China (1/0) | 40/0 | T: 20/20, C: 20/20 | T: 14/6, C: 13/7 | T: 62.5±NS, C: 62.5±NS | NS/NS | Lung-Kidney deficiency |
| Guo, 2008 [39] | China (1/0) | 100/0 | T: 50/50, C: 50/50 | T: 36/14, C: 34/16 | T: 56±NS, C: 55±NS | T: I-8, II-33, III-9; C: I-7, II-35, III-8/NS | NS |
| Hu, 2012 [40] | China (6/0) | 101/0 | T: 51/51, C: 50/50 | T: 34/17, C: 34/16 | T: 65.41±12.23, C: 64.86±11.84 | T:II-26, III-25; C: II-25, III-25/NS | Lung-Kidney deficiency & Blood Stasis with Stagnation of Phlegm |
| Huang, 2005 [41] | China (3/6) | 63/0 | T: 32/32, C: 31/31 | T: 21/11, C: 23/8 | T: 69.5±11.8, C: 68.8±10.6 | T: II-17, III-15; C: II-18, III-13/T: 8.5±3.2, C: 8.3±3.5 | NS |
| Jian, 2012 [42] | China (12/6) | 70/3 (T:2, C:1; poor compliance) | T: 35/33, C: 35/34 | T: 25/10, C: 23/12 | total: 69.46±9.31 | NS/ total: 14.90±6.50 | NS |
| Li, 2012 [43] | China (6/12) | 352/2 (violated the protocol) | T: 176/176, C: 176/174 | T: 122/54, C: 131/43 | T: 66.33±9.63, C: 64.28±9.42 | T: I-14, II-68, III-94; C: I-6, II-80, III-88/T: 8.5±3.2, C: 8.3±3.5 | Lung-Spleen Qi deficiency or Lung-Kidney Qi deficiency or Lung-Kidney Qi-Yin deficiency |
| Liao, 2011 [44] | China (4/0) | 63/0 | T: 30/30, C: 33/33 | T: 21/9, C: 23/10 | T: 37~72, C: 39~70 | NS/ T: 169.56±290.63 (mths), C: 161.07±128.45 (mths) | Lung Qi deficiency |
| Liu, 2009 [45] | China (3/0) | 60/0 | T: 30/30, C: 30/30 | T: 18/12, C: 16/14 | T: 65±NS, C: 64±NS | T: 0-5, I-9, IIA-10, IIB-6; C: 0-6, I-10, IIA-10, IIB-4/T: 17±NS, C: 16±NS | Qi deficiency with Phlegm and Blood Stasis |
| Mao, 2009 [46] | China (3/12) | 108/NS | T: 58 /50, C: 58/50 | T: 42/16, C: 39/11 | T: 64.58±7.07, C: 64.28±7.21 | NS/T: 16.79±6.43, C: 16.72±7.19 | NS |
| Shan, 2011 [47] | China (6/0) | 60/3 (T:1, C:2; not attend an appointment) | T: 30/29, C: 30/28 | T: 23/6, C: 24/4 | T: 66.83±5.85, C: 66.93±4.77 | T: I-6, II-23; C: I-7, II-21/T: 12.59±4.10, C: 13.43±3.79 | NS |
| Xu(2), 2012 [48] | China (3/0) | 90/0 | T: 45/45, C: 45/45 | T: 29/16, C: 32/13 | T: 55.8±NS, C: 56.2±NS | T: I-5, II-26, III-14; C: I-3, II-30, III-12/T: 7.6±NS, C: 6.9±NS | Lung-Spleen deficiency or Lung-Kidney deficiency |
| Xu (1), 2012 [49] | China (3/0) | 40/8 (T:3, C:5; NS) | T: 20/17, C: 20/15 | T: 10/7, C: 7/8 | T: 63.65±9.88, C: 56.93±11.79 | NS/T: 24±10.56, C: 19.73±10.85 | Kidney Qi deficiency or Lung-Spleen Qi deficiency |
| Yu, 2011 [50] | China (3/0) | 98/9 (NS) | T: 44/ 44, C: 43/43 | T 28/16, C: 26/17 | T: 64.11±10.83, C: 65.06±9.84 | T: I-27, II-17; C: I-26, II-17/NS | Qi deficiency with Blood Stasis |
| Zhang (2), 2007 [51] | China (6/12) | 120/0 | T: 60/60, C: 60/60 | T: 38/22, C: 40/20 | T: 56±NS, C: 55±NS | NS/T: 16±NS, C: 14±NS | Lung-Kidney Qi deficiency |
| Zhang (1), 2007 [52] | China (1/0) | 120/0 | T: 60/60, C: 60/60 | T: 38/22, C: 40/20 | T: 56±NS, C: 55±NS | T: I-11, II-36, III-13; C: I-12, II-37, III-11/T: 16±NS, C: 14±NS | NS |
| Zhang, 2011 [53] | China (6/0) | 100/12 [T: 7 (5-cannot tolerate treatment, 2-AE); C:5 (1-acute CVD, 4-AE)] | T: 50/ 43, C: 50/45 | T: 36/7, C: 39/6 | T: 56.20±7.12, C: 55.21±7.01 | NS/T: 16.02±8.96, C: 15.26±9.10 | Spleen-Kidney deficiency with cold |
| Zhao, 2012 [54] | China (3/0) | 58/9 (2-death, 1-CVD, 1-surgical operation, 3-not attend an appointment, 2-unwilling to repeat assessment) | T: 26/26, C: 23/23 | total: 46/3 | T: 80.1±8.9, C: 80.8±7.3 | total: II-10, III-23, IV-16/NS | NS |
| Fan, 2012 [55] | China(3/12) | 180/0 | T: 90/90, C: 90/90 | T: 56/34, C: 57/33 | T:58.6±NS, C: 57.7±NS | T: I-10, II-54, III-26; C: I-11, II-54, III-25/T: 7.8±NS, C: 7.7±NS | Lung-Spleen deficiency or Lung-Kidney deficiency |
| Liang, 2013 [56] | China (3/0) | 80/1 (T: 1- violated the protocol) | T: 40/39, C: 40/40 | T: 32/7, C: 38/2 | T:36~75, C: 48~75 | T: III-25, IV-15; C: III-25, IV-15/T: 15.43±5.68, C: 18.35±6.82 | Lung-Kidney deficiency |
| Lin, 2013 [57] | China (3/0) | 68/0 | T: 34/34, C: 34/34 | T: 25/9, C: 26/8 | T: 65.1±8.2, C: 62.5±7.9 | T: I-8, II-26; C: I-9, II-25/T: 18.7±4.1, C: 20.5±3.8 | NS |
| Peng, 2013 [58] | China (2/0) | 60/5 [T: 2-poor compliance; C:3 (1- poor compliance, 1-not attend an appointment, 1-AE)] | T: 30/28, C: 30/27 | T: 19/9, C: 20/7 | T: 63.07±10.64, C: 62.44±10.11 | T: I-7, II-21; C: I-8, II-29/T: 6.16±3.69, C: 5.96±3.44 | Lung Qi deficiency |
| Yang, 2013 [60] | China (1/0) | 94/0 | T: 54/54, C: 40/40 | T: 32/22 C: 23/17 | T: 62.7±8.6, C: 60.9±7.9 | total: I-NS, II-NS/NS | NS |
| Zeng, 2013 [59] | China (3/0) | 120/0 | T: 60/60, C: 60/60 | T: 33/27 C: 36/24 | T: 61±11, C: 63±12 | NS/NS | NS |

CHM: Chinese Herbal Medicine, RP: routine pharmacotherapy, mths: months, R/A: registration/analysis, M/F: male/female, yrs: years, CM: Chinese Medicine, NS: not stated, T: test group, C: control group, CVD: cerebrovascular disease. AE: acute exacerbation.
