# Supplementary material for: Oral Chinese Herbal Medicine Combined with Pharmacotherapy for Stable COPD: A Systematic Review of Effect on BODE Index and Six Minute Walk Test
Source: PLoS One. 2014 Mar 12;9(3):e91830. doi: 10.1371/journal.pone.0091830 (PMC3951501; doi:10.1371/journal.pone.0091830)
Supplement: Table S3 — Interventions of the 25 studies of CHM plus RP for stable COPD. CHM: Chinese Herbal Medicine, RP: routine pharmacotherapy, Y: yes, N: no, CSRD: COPD Study Group of Chinese Society of Respiratory Disease: Treatment guidelines of COPD, GOLD: Global Initiative for Chronic Obstructive Lung Disease. (DOCX) [file pone.0091830.s004.docx]

**Table S3 Interventions of the 25 studies of CHM plus RP for stable COPD**

| First author, year | Test Group: CHM/dosage: Ingredients (Quality Control: Y/N) | Control Group: RP (same RP also used in Test Group) |
| --- | --- | --- |
| Chen, 2009 [37] | Qi-Wei Du-Qi Tang (decoction), 250 ml bid: *Cornus officinalis* (Shan Zhu Yu), *Dioscorea opposita* (Shan Yao), *Rehmannia glutinosa* (Shu Di Huang), *Paeonia suffruticosa* (Mu Dan Pi), *Alisma plantago-aquatica* (Ze Xie), *Poria cocos* (Fu Ling), *Schisandra chinensis* (Wu Wei Zi), *Astragalus membranaceus* (Huang Qi), *Codonopsis pilosula* (Dang Shen), *Atractylodes macrocephala* (Bai Zhu) (QC: N). | salmeterol 50μg & fluticasone 500μg (inhaled), 1 puff bid |
| Chen, 2012 [36] | Bu-Zhong Yi-Qi Tang (decoction), 150ml, bid: *Astragalus membranaceus* (Huang Qi), *Codonopsis pilosula* (Dang Shen), *Atractylodes macrocephala* (Bai Zhu), *Cimicifuga foetida* (Sheng Ma), *Bupleurum chinense* (Chai Hu), *Angelica sinensis* (Dang Gui), *Citrus tangerina*(Chen Pi), *Glycyrrhiza uralensis* (Gan Cao) (QC: Y). | compound ipratropium bromide solution for inhalation (salbutamol/ipratropium, inhaled) plus ambroxol hydrochloride tablets (oral) plus pulmonary exercise |
| Cui, 2004 [38] | Tong-Fei Mixture (decoction), 1 packet/day, in two doses: *Rehmannia glutinosa* (Sheng Di), *Angelica sinensis* (Dang Gui), *Astragalus membranaceus* (Huang Qi), *Salvia miltiorrhiza* (Dan Shen), *Lilium brownii* (Bai He), *Ophiopogon japonicus* (Mai Men Dong), *Panax ginseng* (Ren Shen), *Pinellia ternata* (Ban Xia), *Fritillaria cirrhosa* (Chuan Bei Mu), *Trichosanthes kirilowii* (Gua Lou), *Poria cocos* (Fu Ling), *Glycyrrhiza uralensis* (Gan Cao), *Perilla frutescens* (Zi Su Zi), *Citrus tangerina* (Jv Hong) (QC: N). | ipratropium bromide aerosol (inhaled) plus oxygen therapy, 2L/min |
| Guo, 2008 [39] | Jian-Fei Capsule, 2-3 caps tid: *Panax ginseng* (Ren Shen)*, Gekko gecko* (Ge Jie)*. Fagopyrum dibotrys* (Jin Qiao Mai)*, Pheretima aspergillum* (Di Long)*, Fritillaria cirrhosa* (Chuan Bei Mu)*, Prunus armeniaca* (Xing Ren)*,* etc. (QC: Y). | theophylline tablets (oral) plus Mucosolvan (oral) plus long-acting β2-agonists (inhaled) |
| Hu, 2012 [40] | 1. Jin-Shui-Bao Capsule, 3 caps (0.33g/cap) tid: cultured *Cordyceps sinensis* mycelia (Dong Chong Xia Cao) (QC: Y); plus: 2. Bu-Fei-Huo-Xue capsule, 4 caps (0.35g/cap) tid: *Astragalus membranaceus* (Huang Qi), *Paeonia veitchii* (Chi Shao), *Psoralea corylifolia* (Bu Gu Zhi) (QC: Y). | salbutamol sulphate aerosol (inhaled) |
| Huang, 2005 [41] | 1. Yu-Ping-Feng Granule, 5g tid: *Astragalus membranaceus* (Huang Qi)*, Atractylodes macrocephala* (Bai Zhu)*, Saposhnikovia divaricata* (Fang Feng) (QC: Y); plus: 2. Bai-Ling Capsule, 1g tid: cultured *Cordyceps sinensis* mycelia (Dong Chong Xia Cao) (QC: Y); plus: 3. Jian-Pi Yi-Fei Granule, 10g tid: *Panax ginseng* (Ren Shen)*, Atractylodes macrocephala* (Bai Zhu)*, Poria cocos* (Fu Ling)*, Ophiopogon japonicus* (Mai Men Dong)*, Morus alba* (Sang Bai Pi)*, Astragalus membranaceus* (Huang Qi) (QC: Y). | treated according to the CSRD |
| Jian, 2012 [42] | Ping-Chuan Capsule, 4 caps (1.5g/cap) tid: *Panax ginseng* (Ren Shen)*, Gekko gecko* (Ge jie)*, Panax pseudo-ginseng* (Tian Qi), etc. (QC: Y). | ipratropium bromide aerosol (inhaled), 1 puff bid |
| Li, 2012 [43] | 1. Bu-Fei Jian-Pi Granule (Lung-Spleen Qi deficiency ), 11.49g bid: *Astragalus membranaceus* (Huang Qi)*, Codonopsis pilosula* (Dang Shen)*, Atractylodes macrocephala* (Bai Zhu)*, Poria cocos* (Fu Ling)*, Fritillaria cirrhosa* (Chuan Bei Mu) (QC: Y); or 2. Bu-Fei Yi-Shen Granule (Lung-Kidney Qi deficiency), 12.75g bid: *Panax ginseng* (Ren Shen), *Astragalus membranaceus* (Huang Qi), *Lycium barbarum* (Gou Qi Zi), *Cornus officinalis* (Shan Zhu Yu), *Epimedium brevicornum* (Yin Yang Huo) (QC: Y); or 3. Yi-Qi Zi-Shen granule (Lung-Kidney Qi and Yin deficiency), 15.48g bid: *Panax ginseng* (Ren Shen), *Polygonatum sibiricum* (Huang Jing), *Rehmannia glutinosa* (Shu Di Huang), *Ophiopogon japonicus* (Mai Men Dong), *Schisandra chinensis* (Wu Wei Zi) (QC: Y). | I: active reduction of risk factors; add short-acting bronchodilator (when needed), i.e. albuterol sulphate (Ventolin 100μg/dose), dosing: 1–2 puffs each time, up to 8–12 puffs a day; II: add regular treatment with one long-acting bronchodilator, i.e. formoterol fumarate dehydrate (Oxis Turbuhaler 4.5μg/dose), dosing: 1 puff each time, twice daily; III: add inhaled glucocorticosteroids if repeated exacerbations, i.e. salmeterol/fluticasone propionate (Seretide 50/250 μg/dose); dosing: 1 puff each time, twice daily |
| Liao, 2011 [44] | Yu-Ping-Feng Granule, 4g tid: *Astragalus membranaceus* (Huang Qi)*, Atractylodes macrocephala* (Bai Zhu)*, Saposhnikovia divaricata* (Fang Feng) (QC: Y). | RP unspecified |
| Liu, 2009 [45] | 1. Yi-Qi Huo-Xue Tong-Luo Formula (decoction), 100ml/d, in two doses: *Astragalus membranaceus* (Huang Qi)*, Codonopsis pilosula* (Dang Shen)*, Poria cocos* (Fu Ling)*, Atractylodes macrocephala* (Bai Zhu)*, Fritillaria cirrhosa* (Bei Mu)*, Pheretima aspergillum* (Di Long)*, Salvia miltiorrhiza* (Dan Shen)*, Asarum heterotropoides* (Xi Xin)*, Brassica alba* (Bai Jie Zi)*, Fluoritum* (Zi Shi Ying)*, Rhodiola sacra* (Hong Jing Tian) (QC: Y); plus: 2. Bai-Ling Capsule, 5-10 caps (0.2g/cap) bid: cultured *Cordyceps sinensis* mycelia (Dong Chong Xia Cao) (QC: Y); or 3. Jin-Shui-Bao Capsule, 5caps (0.33g/cap), bid: cultured *Cordyceps sinensis* mycelia (Dong Chong Xia Cao) (QC: Y). | treated according to the GOLD (stage 0-II) |
| Mao, 2009 [46] | Bai-Ling Capsule, 5 caps (0.2g/cap), tid: cultured *Cordyceps sinensis* mycelia (Dong Chong Xia Cao) (QC: Y). | compound drug: aminophylline 75mg& bromhexine 15mg& chlorphenamine115mg tablets (oral), 1.5mg tid |
| Shan, 2011 [47] | Pei-Tu Sheng-Jin Formula (decoction), 1 packet per day: *Codonopsis pilosula* (Dang Shen), *Cayratia japonica* (Wu Zhua Long), *Poria cocos* (Fu Ling), *Atractylodes macrocephala* (Bai Zhu), *Dioscorea opposite* (Huai Shan Yao), *Schisandra chinensis* (Wu Wei Zi), *Tussilago farfara* (Kuan Dong Hua), *Prunus armeniaca* (Bei Xing Ren), *Prunus persica* (Tao Ren), *Perilla frutescens* (Zi Su Zi), *Gallus domesticus* (Ji Nei Jin), *Glycyrrhiza uralensis* (Gan Cao) (QC: N). | theophylline sustained-release tablets (oral), 0.2g q12h |
| Xu(2), 2012 [48] | Gu-Jin Gao (decoction), 15ml, bid: *Astragalus membranaceus* (Huang Qi)*, Pseudostellaria heterophylla* (Tai Zi Shen)*, Atractylodes macrocephala* (Bai Zhu)*, Dioscorea opposite* (Shan Yao)*, Poria cocos* (Fu Ling)*, Citrus tangerina* (Chen Pi)*, Amomum villosum* (Sha Ren)*, Saposhnikovia divaricata* (Fang Feng)*, Glehnia littoralis (*Su Tiao Shen)*, Ophiopogon japonicus* (Mai Men Dong)*, Cistanche salsa* (Rou Cong Rou)*, Epimedium grandiflorum* (Yin Yang Huo)*, Acanthopanax gracilistylus* (Wu Jia Pi)*, Gynostemma pentaphyllum* (Jiao Gu Lan)*, Houttuynia cordata* (Yu Xing Cao)*, Lepidium apetalum* (Ting Li Zi)*, Salvia miltiorrhiza* (Zi Dan Shen)*, Glycyrrhiza uralensis* (Gan Cao)*, Equue asinus* (E Jiao)*,* Sugar (Yi Tang), etc. (QC: N). | treated according to CSRD |
| Xu (1), 2012 [49] | 1. Wen-Shen Yi-Qi granule (Kidney Qi deficiency) 1 bag tid: *Astragalus membranaceus* (Huang Qi), *Curcuma zedoaria* (E zhu), *Fluoritum* (Zi Shi Ying), *Cuscuta chinensis* (Tu Si Zi), etc. (QC: N); or 2. Li-Fei Jian-Pi Granule (Lung-Spleen Qi deficiency), 1 bag tid: *Codonopsis pilosula* (Dang Shen), *Atractylodes lancea* (Cang Zhu), *Scutellaria baicalensis* (Huang Qin), *Arisaema heterophyllum* (Dan Xing), etc.(QC: N). | treated according to the CSRD: Stage 1: Ventolin inhaled; Stage 2: inhale formoterol/budesonide or tiotropium; Stage 3: inhaled salmeterol/fluticasone or formoterol/ budesonide or tiotropium |
| Yu, 2011 [50] | Ge-Jie Ding-Chuan Capsule, 3 caps tid: *Gekko gecko* (Ge Jie), *Perilla frutescens* (Zi Su Zi), *Trichosanthes kirilowii* (Gua Lou Zi), *Prunus armeniaca* (Xing Ren), *Ephedra sinica* (Ma Huang), *Crystalline gypsum* (Shi Gao), *Glycyrrhiza uralensis* (Gan Cao), *Aster tartaricus* (Zi Wan), *Amyda sinensis* (Bie Jia), *Scutellaria baicalensis* (Huang Qin), *Ophiopogon japonicus* (Mai Men Dong), *Coptis chinensis* (Huang Lian), *Lilium brownii* (Bai He),*Crystalline gypsum* (Shi Gao), etc. (QC: N). | treated according to GOLD: Stage 1: no treatment; Stage 2: theophylline sustained-release tablets: 0.1g, bid |
| Zhang (2), 2007 [51] | Bailing Capsule 5 caps (0.2g/cap) tid: cultured *Cordyceps sinensis* mycelia (Dong Chong Xia Cao) (QC: Y). | ipratropium bromide aerosol (inhaled), 2 puffs tid |
| Zhang (1), 2007 [52] | Basic Formula (decoction), 100ml/day, in 2 doses (day & night): *Panax ginseng* (Ren Shen)*, Gekko gecko* (Ge Jie)*, Fagopyrum dibotrys* (Jin Qiao Mai)*, Prunus armeniaca* (Xing Ren)*, Pheretima aspergillum* (Di Long)*, Fritillaria cirrhosa* (Chuan Bei) (QC: N). | theophylline tablets (oral) plus Mucosolvan (oral) plus long-acting β2-agonists (inhaled) |
| Zhang, 2011 [53] | 1. Yu-Ping-Feng San (decoction): *Astragalus membranaceus* (Huang Qi)*, Atractylodes macrocephala* (Bai Zhu)*, Saposhnikovia divaricata* (Fang Feng) (QC: N); plus: 2. Jin-Shui-Liu-Jun Jian (decoction): *Angelica sinensis* (Dang Gui)*, Rehmannia glutinosa* (Shu Di Huang)*, Citrus tangerina* (Chen Pi)*, Pinellia ternata* (Ban Xia)*, Poria cocos* (Fu Ling)*, Glycyrrhiza uralensis* (Gan Cao) (QC: N). | salmeterol 50μg & fluticasone 500μg (inhaled) |
| Zhao, 2012 [54] | Chinese Yam-Epimedium Mixture (decoction), 80ml bid: *Dioscorea opposita* (Shan Yao)*, Epimedium brevicornum* (Xian Lin Pi) (QC: Y). | placebo decoction plus western medicine plus pulmonary rehabilitation |
| Fan, 2012 [55] | Bu-Fei-Jian-Pi-Yi-Shen Formula (decoction), 200ml bid: *Astragalus membranaceus* (Huang Qi), *Codonopsis pilosula* (Dang Shen), *Atractylodes macrocephala* (Bai Zhu)*, Poria cocos* (Fu Ling)*, Saposhnikovia divaricata* (Fang Feng), *Glehnia littoralis* (Su Tiao Shen)*, Gynostemma pentaphyllum* (Jiao Gu Lan)*, Houttuynia cordata* (Yu Xing Cao)*, Citrus tangerina* (Chen Pi)*, Epimedium brevicornum* (Yin Yang Huo), *Acanthopanax gracilistylus* (Wu Jia Pi)*, Salvia miltiorrhiza* (Dan Shen), *Glycyrrhiza uralensis* (Gan Cao) (QC: Y). | treated according to CSRD |
| Liang, 2013 [56] | Run-Fei-Jian-Pi-Bu-Shen Formula (decoction), 100ml bid: *Glehnia littoralis* (Su Tiao Shen)*,* *Ophiopogon japonicus* (Mai Men Dong), *Lycium chinense* (Di Gu Pi), *Morus alba* (Sang Bai Pi)*,* *Panax ginseng* (Ren Shen)*, Poria cocos* (Fu Ling)*, Morinda officinalis* (Ba Ji Tian), *Pheretima aspergillum* (Di Long)*, Atractylodes macrocephala* (Bai Zhu)*, Ligusticum chuanxiong* (Chuan Xiong), *Ostrea rivularis* (Mu Li), *Buthus martensi* (Quan Xie), *Paris polyphylla* (Zi He Che) (QC: N). | salmeterol 50μg & fluticasone 500μg (inhaled), 1 puff bid |
| Lin, 2013 [57] | Bu-Fei Formula plus Ling-Gui-Zhu-Gan Formula (decoction), 400ml/day, in 2 doses (day & night): *Astragalus membranaceus* (Huang Qi), *Codonopsis pilosula* (Dang Shen), *Atractylodes macrocephala* (Bai Zhu)*, Poria cocos* (Fu Ling)*, Salvia miltiorrhiza* (Dan Shen), *Glycyrrhiza uralensis* (Gan Cao), *Citrus tangerina* (Chen Pi)*, Pinellia ternata* (Ban Xia)*, Rehmannia glutinosa* (Shu Di Huang), *Schisandra chinensis* (Wu Wei Zi), *Aster tartaricus* (Zi Wan), *Perilla frutescens* (Zi Su Zi), *Cinnamomum cassia* (Gui Zhi) (QC: N). | salmeterol 50μg &fluticasone 250μg (inhaled), 1 puff bid plus theophylline sustained-release capsule (oral), 100-200mg bid |
| Peng, 2013 [58] | Bu-FeiFormula No.1 (decoction), 300ml/day, in 2 doses (day & night): *Astragalus membranaceus* (Huang Qi), *Acanthopanax gracilistylus* (Wu Jia Pi)*, Polygonatum sibiricum* (Huang Jing), *Agrimonia pilosa* (Xian He Cao) (QC: Y). | theophylline tablets (oral), 0.2g q12h plus pulmonary rehabilitation |
| Yang, 2013 [60] | Tong-Qi-Pai-Yong Formula (decoction), 300ml/day, in 2 doses (day & night): *Astragalus membranaceus* (Huang Qi), *Panax ginseng* (Ren Shen)*, Atractylodes macrocephala* (Bai Zhu)*, Citrus tangerina* (Chen Pi)*, Pinellia ternata* (Ban Xia)*, Poria cocos* (Fu Ling)*, Glycyrrhiza uralensis* (Gan Cao), *Ophiopogon japonicus* (Mai Men Dong), *Paeonia veitchii* (Chi Shao), *Dallbergia odorifera* (Jiang Xiang) (QC: N). | salmeterol 50μg & fluticasone 500μg (inhaled), 1 puff bid |
| Zeng, 2013 [59] | Modified Si-Jun-Zi Granule, NS: *Codonopsis pilosula* (Dang Shen), *Astragalus membranaceus* (Huang Qi), *Poria cocos* (Fu Ling), *Cordyceps sinensis mycelia* (Dong Chong Xia Cao) (QC: Y). | RP unspecified plus pulmonary rehabilitation |

CHM: Chinese Herbal Medicine, RP: routine pharmacotherapy, Y: yes, N: no, CSRD: COPD Study Group of Chinese Society of Respiratory Disease: Treatment guidelines of COPD, GOLD: Global Initiative for Chronic Obstructive Lung Disease.
