# Supplementary material for: Oral Chinese Herbal Medicine Combined with Pharmacotherapy for Stable COPD: A Systematic Review of Effect on BODE Index and Six Minute Walk Test
Source: PLoS One. 2014 Mar 12;9(3):e91830. doi: 10.1371/journal.pone.0091830 (PMC3951501; doi:10.1371/journal.pone.0091830)
Supplement: Table S4 — Results for individual studies: BODE Index (MD, 95%CI) for the CHM plus RP groups (T) and the RP groups (C) at baseline and end of treatment. *Significant difference, MD: mean difference, CI: confidence interval, CHM: Chinese Herbal Medicine, RP: routine pharmacotherapy, T: test group, C: control group, EoT: end of treatment, mths: months, Ipra.: ipratropium (inhaled), Salb.: salbutamol (inhaled), Theo.(+p.r.): oral theophylline (plus pulmonary rehabilitation), Theo.: theophylline (oral), Salm./Flu.: salmeterol/fluticasone (inhaled), RP (guidelines): routine pharmacotherapy (adjusted for severity according to guidelines), RP(+ p.r.): routine pharmacotherapy (pharmacotherapy plus pulmonary rehabilitation). (DOCX) [file pone.0091830.s005.docx]

# Table S4 Results for individual studies: BODE Index (MD, 95%CI) for the CHM plus RP groups (T) and the RP groups (C) at baseline and end of treatment

| First author, year | Intervention (Duration) | T vs C at baseline | C: baseline vs EoT | T: baseline vs EoT | T vs C at EoT |
| --- | --- | --- | --- | --- | --- |
| Jian,2012 [42] | CHM+Ipra.(12mths) | Not estimable | Not estimable | Not estimable | -0.68 [-1.10, -0.26]* |
| Hu,2012 [40] | CHM+Salb.(6mths) | 0.02 [-1.11, 1.15] | -0.47 [-1.61, 0.67] | -1.70 [-2.76, -0.64]* | -1.21 [-2.29, -0.13]* |
| Peng,2013 [58] | CHM+Theo.(+p.r). (2mths) | 0.07 [-0.91, 1.05] | -0.15 [-1.03, 0.73] | -0.25 [-1.25, 0.75] | -0.03 [-0.93, 0.87] |
| Shan,2011 [47] | CHM+Theo.(6mths) | 0.20 [-0.46, 0.86] | -0.07 [-0.76, 0.62] | -1.35 [-1.99, -0.71]* | -1.08 [-1.76, -0.40]* |
| Chen,2009 [37] | CHM+Salm./Flu.(3mths) | -0.01 [-1.26, 1.24] | -1.44 [-2.65, -0.23]* | -2.64 [-3.82, -1.46]* | -1.21 [-2.34, -0.08]* |
| Yang,2013 [60] | CHM+Salm./Flu.(1mth) | -0.10 [-0.59, 0.39] | -0.27 [-0.78, 0.24] | -0.78 [-1.21, -0.35] * | -0.61 [-1.07, -0.15] * |
| Xu(1),2012 [49] | CHM+RP (guidelines)(3mths) | 0.61 [-0.85, 2.07] | -0.86 [-2.23, 0.51] | -0.82 [-2.16, 0.52] | 0.65 [-0.59, 1.89] |
| Yu,2011 [50] | CHM+RP (guidelines)(3mths) | 0.00 [-0.68, 0.68] | -0.61 [-1.40, 0.18] | -1.39 [-1.98, -0.80]* | -0.78 [-1.50, -0.06]* |
| Zhao,2012 [54] | CHM+RP (+ p.r.)(3mths) | 0.00 [-1.60, 1.60] | 0.30 [-1.64, 2.24] | -2.10 [-3.49, -0.71]* | -2.40 [-4.17, -0.63]* |

*Significant difference, MD: mean difference, CI: confidence interval, CHM: Chinese Herbal Medicine, RP: routine pharmacotherapy, T: test group, C: control group, EoT: end of treatment, mths: months, Ipra.: ipratropium (inhaled), Salb.: salbutamol (inhaled), Theo.(+p.r.): oral theophylline (plus pulmonary rehabilitation), Theo.: theophylline (oral), Salm./Flu.: salmeterol/fluticasone (inhaled), RP (guidelines): routine pharmacotherapy (adjusted for severity according to guidelines), RP(+ p.r.): routine pharmacotherapy (pharmacotherapy plus pulmonary rehabilitation).
