# Supplementary material for: Oral Chinese Herbal Medicine Combined with Pharmacotherapy for Stable COPD: A Systematic Review of Effect on BODE Index and Six Minute Walk Test
Source: PLoS One. 2014 Mar 12;9(3):e91830. doi: 10.1371/journal.pone.0091830 (PMC3951501; doi:10.1371/journal.pone.0091830)
Supplement: Table S5 — Results for individual studies: 6MWT/D (MD, 95%CI, meters) for the CHM plus RP groups (T) and the RP groups (C) at baseline and end of treatment. *Significant difference, CHM: Chinese Herbal Medicine, RP: routine pharmacotherapy, MD: mean difference, mths: months, CI: confidence interval, T: test group, C: control group, EoT: end of treatment, Salm./Flu.: salmeterol/fluticasone (inhaled), Theo.(+p.r.): oral theophylline (plus pulmonary rehabilitation), Theo.: theophylline (oral), Muc.: Mucosolvan (oral), LABA: long-acting β2 agonist (inhaled), Ami./Bro./Chlo.: compound oral medication which contained aminophylline, bromhexine and chlorphenamine, Ipra.: ipratropium (inhaled), oxy.: oxygen therapy, Salb./Ipra.: salbutamol/ipratropium (inhaled), exe.: pulmonary exercise, RP(guidelines): routine pharmacotherapy (adjusted for severity according to guidelines), RP(+ p.r.): routine pharmacotherapy (pharmacotherapy plus pulmonary rehabilitation). (DOCX) [file pone.0091830.s006.docx]

# Table S5 Results for individual studies: 6MWT/D (MD, 95%CI, meters) for the CHM plus RP groups (T) and the RP groups (C) at baseline and end of treatment

| First author, year | Intervention (Duration) | T vs C at baseline | C: baseline vs EoT | T: baseline vs EoT | T vs C at EoT |
| --- | --- | --- | --- | --- | --- |
| Zhang, 2011 [53] | CHM+Salm./Flu.(6mths) | 2.06 [-12.63, 16.75] | 5.06 [-10.36, 20.48] | 28.27 [8.02, 48.52]* | 25.27 [4.48, 46.06]* |
| Chen, 2009 [37] | CHM+Salm./Flu.(3mths) | 3.85 [-31.46, 39.16] | 37.05 [1.12, 72.98]* | 80.84 [45.49, 116.19]* | 47.64 [11.67, 83.61]* |
| Peng, 2013 [58] | CHM+Theo.(+ p.r.)(2mths) | -1.51 [-33.37, 30.35] | 2.63 [-30.09, 35.35] | 6.47 [-27.02, 39.96] | 2.33 [-31.98, 36.64] |
| Guo, 2008 [39] | CHM+Theo.&Muc.&LABA(1mth) | -6.00 [-37.75, 25.75] | 60.00 [30.35, 89.65]* | 118.00 [87.01, 148.99]* | 52.00 [22.60, 81.40]* |
| Zhang(1), 2007 [52] | CHM+Theo.&Muc.&LABA(1mth) | -4.00 [-32.27, 24.27] | 70.00 [42.43, 97.57]* | 112.00 [83.36, 140.64]* | 38.00 [10.05, 65.95]* |
| Mao, 2009 [46] | CHM+Ami./Bro./Chlo.(3mths) | 2.80 [-21.34, 26.94] | 17.50 [-6.47, 41.47] | 72.30 [48.54, 96.06]* | 57.60 [34.02, 81.18]* |
| Cui, 2004 [38] | CHM+Ipra.(+oxy.)(1mth) | 1.00 [-23.25, 25.25] | 6.00 [-16.07, 28.07] | 124.00 [87.02, 160.98]* | 119.00 [83.41, 154.59]* |
| Zhang(2), 2007 [51] | CHM+Ipra.(6mths) | -4.00 [-31.56, 23.56] | 23.00 [-4.21, 50.21] | 62.00 [34.08, 89.92]* | 35.00 [7.43, 62.57]* |
| Chen, 2012 [36] | CHM+Salb./Ipra.&Muc.(+exe.)(6mths) | -3.72 [-15.70, 8.26] | 94.20 [79.66, 108.74]* | 163.00 [153.76, 172.24]* | 65.08 [52.71, 77.45]* |
| Xu(2), 2012 [48] | CHM+RP(guidelines)(3mths) | -5.20 [-42.95, 32.55] | -28.40 [-64.77, 7.97] | 32.10 [-6.48, 70.68] | 55.30 [18.07, 92.53]* |
| Xu (1), 2012 [49] | CHM+RP(guidelines)(3mths) | -37.88 [-82.63, 6.87] | 5.90 [-33.31, 45.11] | 28.82 [-19.52, 77.16] | -14.93 [-58.19, 28.33] |
| Huang, 2005 [41] | CHM+RP(guidelines)(3mths) | -1.36 [-24.18, 21.46] | 22.10 [-1.04, 45.24] | 47.56 [25.18, 69.94]* | 24.07 [1.36, 46.78]* |
| Liu, 2009 [45] | CHM+RP(guidelines)(3mths) | 3.00 [-21.05, 27.05] | 35.00 [8.51, 61.49]* | 98.00 [68.87, 127.13]* | 66.00 [34.83, 97.17]* |
| Fan, 2012 [55] | CHM+RP(guidelines)(3mths) | -0.90 [-27.14, 25.34] | -24.10 [-49.34, 1.14] | 33.20 [5.92, 60.48]* | 56.40 [30.08, 82.72]* |
| Liao, 2011[44] | CHM+RP(4mths) | -3.00 [-25.72, 19.72] | 32.00 [6.84, 57.16]* | 87.00 [61.80, 112.20]* | 52.00 [24.57, 79.43]* |
| Zhao, 2012 [54] | CHM+RP (+ p.r.)(3mths) | 20.00 [-66.35, 106.35] | -21.40 [-125.84, 83.04] | 64.10 [-11.65, 139.85] | 105.50 [9.64, 201.36]* |
| Zeng, 2013 [59] | CHM+RP(+ p.r.)(3mths) | -0.66 [-13.59, 12.27] | 75.34 [60.49, 90.19]* | 232.02 [216.19, 247.85]* | 156.02 [138.58, 173.46]* |

*Significant difference, CHM: Chinese Herbal Medicine, RP: routine pharmacotherapy, MD: mean difference, mths: months, CI: confidence interval, T: test group, C: control group, EoT: end of treatment, Salm./Flu.: salmeterol/fluticasone (inhaled), Theo.(+p.r.): oral theophylline (plus pulmonary rehabilitation), Theo.: theophylline (oral), Muc.: Mucosolvan (oral), LABA: long-acting β2 agonist (inhaled), Ami./Bro./Chlo.: compound oral medication which contained aminophylline, bromhexine and chlorphenamine, Ipra.: ipratropium (inhaled), oxy.: oxygen therapy, Salb./Ipra.: salbutamol/ipratropium (inhaled), exe.: pulmonary exercise, RP(guidelines): routine pharmacotherapy (adjusted for severity according to guidelines), RP(+ p.r.): routine pharmacotherapy (pharmacotherapy plus pulmonary rehabilitation).
